# Age-related shift in LTD is dependent on neuronal adenosine A_2A_ receptors interplay with mGluR5 and NMDA receptors

**DOI:** 10.1038/s41380-018-0110-9

**Published:** 2018-06-27

**Authors:** Mariana Temido-Ferreira, Diana G. Ferreira, Vânia L. Batalha, Inês Marques-Morgado, Joana E. Coelho, Pedro Pereira, Rui Gomes, Andreia Pinto, Sara Carvalho, Paula M. Canas, Laetitia Cuvelier, Valerie Buée-Scherrer, Emilie Faivre, Younis Baqi, Christa E. Müller, José Pimentel, Serge N. Schiffmann, Luc Buée, Michael Bader, Tiago F. Outeiro, David Blum, Rodrigo A. Cunha, Hélène Marie, Paula A. Pousinha, Luísa V. Lopes

**Affiliations:** 1 Instituto de Medicina Molecular, Faculdade de Medicina de Lisboa, Universidade de Lisboa, 1649-028 Lisbon, Portugal; 2 Department of Experimental Neurodegeneration, Center for Nanoscale Microscopy and Molecular Physiology of the Brain, Center for Biostructural Imaging of Neurodegeneration, University Medical Center Göttingen, Waldweg 33, 37073 Göttingen, Germany; 3 Department of Pharmacology and Therapeutics, Faculty of Medicine, University of Porto, Porto, Portugal; 4 MedInUP—Center for Drug Discovery and Innovative Medicines, University of Porto, 4200-450 Porto, Portugal; 5 Laboratory of Neuropathology, Department of Neurosciences, Hospital de Santa Maria, CHLN, EPE, 1649-035 Lisbon, Portugal; 6 Faculdade de Ciências da Universidade de Lisboa, 1749-016 Lisbon, Portugal; 7 CNC-Center for Neuroscience and Cell Biology, University of Coimbra, 3004-504 Coimbra, Portugal; 8 Faculty of Medicine, University of Coimbra, 3004-504 Coimbra, Portugal; 9 Laboratory of Neurophysiology, ULB Neuroscience Institute, Université Libre de Bruxelles (ULB), 1070 Brussels, Belgium; 10 Université de Lille, Institut National de la Santé et de la Recherche Medicale (INSERM), CHU Lille, UMR-S 1172 JPArc, “Alzheimer & Tauopathie”, LabEx DISTALZ, Lille, France; 11 PharmaCenter Bonn, Pharmazeutische Chemie I, Pharmazeutisches Institut, University of Bonn, Bonn, Germany; 12 Department of Chemistry, Faculty of Science, Sultan Qaboos University, PO Box 36, Postal Code 123 Muscat, Oman; 13 Max-Delbrück-Center for Molecular Medicine (MDC), 13125 Berlin, Germany; 14 Charité-University Medicine, 10117 Berlin, Germany; 15 Institute of Biology, University of Lübeck, 23652 Lübeck, Germany; 16 Max Planck Institute for Experimental Medicine, 37075 Göttingen, Germany; 17 CEDOC, Chronic Diseases Research Center, NOVA Medical School, Faculdade de Ciências Médicas, Universidade NOVA de Lisboa, 1150-082 Lisbon, Portugal; 18 Institute of Neuroscience, The Medical School, Newcastle University, Framlington Place, Newcastle Upon Tyne, NE2 4HH United Kingdom; 19 Université Côte d’Azur, CNRS UMR7276, IPMC, 06560 Valbonne, France

**Keywords:** Neuroscience, Biochemistry

## Abstract

Synaptic dysfunction plays a central role in Alzheimer’s disease (AD), since it drives the cognitive decline. An association between a polymorphism of the adenosine A_2A_ receptor (A_2A_R) encoding gene—*ADORA2A*, and hippocampal volume in AD patients was recently described. In this study, we explore the synaptic function of A_2A_R in age-related conditions. We report, for the first time, a significant overexpression of A_2A_R in hippocampal neurons of aged humans, which is aggravated in AD patients. A similar profile of A_2A_R overexpression in rats was sufficient to drive age-like memory impairments in young animals and to uncover a hippocampal LTD-to-LTP shift. This was accompanied by increased NMDA receptor gating, dependent on mGluR5 and linked to enhanced Ca^2+^ influx. We confirmed the same plasticity shift in memory-impaired aged rats and APP/PS1 mice modeling AD, which was rescued upon A_2A_R blockade. This A_2A_R/mGluR5/NMDAR interaction might prove a suitable alternative for regulating aberrant mGluR5/NMDAR signaling in AD without disrupting their constitutive activity.

## Introduction

Synaptic dysfunction plays a central role in Alzheimer’s disease (AD), since it drives the cognitive decline [1]. In age-related neurodegeneration, cognitive decline has a stronger correlation to early synapse loss than neuronal loss in patients [2]. Despite the many clinical trials conducted to identify drug targets that could reduce protein toxicity in AD, such targets and such strategies proven unsuccessful. Therefore, efforts focused on identifying the early mechanisms of disease pathogenesis, driven or exacerbated by the aging process, may prove more relevant to slow the progression rather than the current disease-based models.

The array of synaptic proteins is complex and the mechanisms underlying excitatory synaptic transmission are finely tuned by synaptic activity. The activation of *N*-methyl-d-aspartate (NMDA) receptors plays a pivotal role, because it can induce either long-term potentiation (LTP) or long-term depression (LTD), depending on the extent of the resultant intracellular [Ca^2+^] rise in the dendritic spines and the downstream activation of specific intracellular cascades [3]. Indeed, the Aβ-triggered synaptic failure involves the removal of AMPA receptors from the synaptic membrane and the degradation of PSD95 protein at glutamatergic synapses [4, 5]. In addition to NMDA receptors and AMPA receptors, an involvement of the metabotropic glutamate receptors (mGlu receptors) in Aβ mediated synaptic dysfunction has been suggested [6]. Shankar and colleagues [6] demonstrated that different sources of Aβ (synthetic, extracted from human brain or from cells) can facilitate mGlu receptor-mediated LTD and can inhibit LTP leading to a reduced dendritic spine density.

The role of LTP has been extensively studied in learning and memory [7–9]. However, much less is known about LTD and memory, either in physiological or pathological conditions. LTD is defined as a long-lasting weakening of a synapse in response to a repeated low-frequency stimulation [10, 11], being required for consolidation of hippocampal-dependent spatial memory [12]. The trigger for inducing activity-dependent LTD is predominantly an increase in postsynaptic calcium (Ca^2+^). Since postsynaptic rises in Ca^2+^ are implicated in the induction of both LTP and LTD [13], it is widely accepted that larger rises in intracellular Ca^2+^ result in LTP induction, while more modest increases lead to LTD induction [14]. Some authors report increased susceptibility to LTD during aging [15], whereas others fail to observe alterations in LTD magnitude in aged animals [16]. These discrepancies can be explained by differences in animal strain, stimulation pattern or Ca^2+^/Mg^2+^ ratio. Accordingly, age-related differences in LTD induction could be rescued by manipulating the extracellular Ca^2+^/Mg^2+^ ratio, consistent with the idea that changes in Ca^2+^ regulation with advanced age may trigger increased susceptibility to LTD [15, 17, 18]. However, the mechanisms leading to calcium alterations in LTD during normal aging and age-related diseases remain mostly unexplored. Recently, an association between a polymorphism of the adenosine A_2A_ receptor (A_2A_R) encoding gene—*ADORA2A*, with hippocampal volume (synaptic loss) in mild cognitive impairment and AD was reported [19]. This polymorphism occurs in a non-coding region, upstream to the coding sequence and it was just suggested, but not studied, that it could imply alterations in A_2A_R expression.

There is compelling evidence from animal models of a cortical and hippocampal upsurge of A_2A_R in glutamatergic synapses upon aging and AD [20–26]. Such A_2A_R overactivation induces glutamate release via PKA/cAMP/CREB signaling [23, 25, 27, 28], calcium influx [29] and leads to hippocampus-dependent cognitive deficits [30, 31]. Conversely, the blockade of A_2A_R, with either caffeine or more selective antagonists (SCH 58261, KW6002, or MSX-3), prevents hippocampus-dependent memory deficits and LTP impairments in aged animals [32, 33] and in several AD models [34–37]. Furthermore, knocking-out A_2A_R can rescue stress and AD-related synaptic dysfunction [38, 39]. Accordingly, in humans, several epidemiological studies have shown that regular caffeine consumption attenuates memory disruption during aging and decreases the risk of developing memory impairments in AD patients [34, 40–43]. Altogether, these data suggest that A_2A_R might be a good candidate as trigger to synaptic dysfunction in aging and AD.

We now explored the role of A_2A_R in hippocampal function in age-related conditions. We showed a significant upsurge of A_2A_R in hippocampal neurons of aged humans, a phenotype aggravated in AD patients. Increased expression of A_2A_R driven by the CaMKII promoter selectively in rat forebrain neurons was sufficient to mimic aging-like memory impairments and to uncover an LTD-to-LTP shift in the hippocampus. This shift was due to an increased NMDA receptor gating and associated to increased Ca^2+^ influx. We identified the mGluR5-NMDAR interplay as key player in the observed A_2A_R-induced synaptic dysfunction. Importantly, the same LTD-to-LTP shift was observed in memory-impaired aged rats and APP/PS1 mice modeling AD, a phenotype rescued upon A_2A_R blockade.

We not only prove that A_2A_R overexpression in young animals is sufficient to drive age-like synaptic impairments, but also explored this newly found interaction as a suitable alternative for regulating aberrant mGluR5/NMDA signaling without disrupting their constitutive activity. Due to the aberrant A_2A_R signaling in pathological conditions (reviewed in ref. [44]), their blockade is particularly relevant for long-term therapies, since the alternative option of targeting directly either mGluR5 or NMDAR interferes with basal neuronal function and memory, as these proteins are crucial components of the postsynaptic density.

## Results

### Increased levels of A_2A_R in human aged and Alzheimer’s disease (AD) brain

**Fig. 1:**
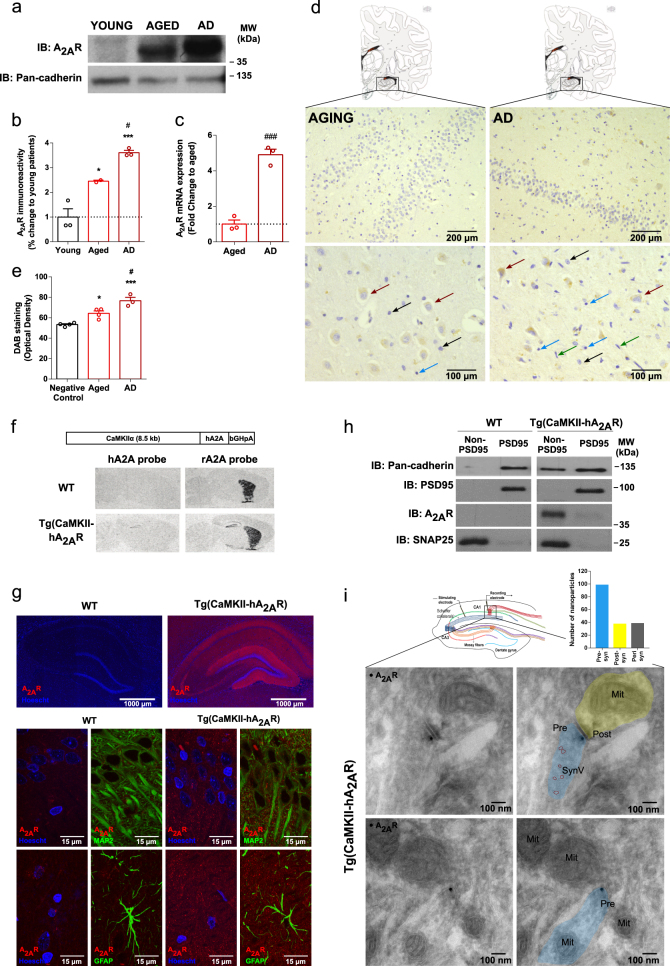
Increased levels of A_2A_R in human aged and Alzheimer’s disease (AD) brain. **a** Representative image of the western blot for A_2A_R in human prefrontal cortex and the internal control Pan-cadherin. **b** A_2A_R immunoreactivity in young, aged, and AD human cortex (*p < 0.05, ***p < 0.001 comparing to young subjects, ^#^*p* < 0.05 comparing to aged subjects, one-way ANOVA followed by a Tukey’s multiple comparisons post hoc test) (*n* = 3, 2, and 3, respectively). **c** Increase in A_2A_R mRNA in AD human brain when compared with age-matched control subjects (^###^*p* < 0.001 comparing to healthy age-matched subjects, unpaired *t*-test) (*n* = 3). **d** In human AD and age-matched control hippocampal sections, positive staining for A_2A_R is present (scale bar: 200 µm). Within hippocampus, A_2A_R upsurge is neuronal specific, since positive labeling is observed in neurons (brown arrows; cells with large hypochromatic nucleus with nucleolar inclusions), while in astrocytes (black arrows; astrocytic nuclei typically have pale, finely granular chromatin patterns and relatively small or indistinct nucleoli), oligodendrocytes (blue arrows; characterized by small, round, relatively dark nuclei) and microglia (green arrows; cells with rod-shaped and often irregularly contoured nuclei) A_2A_R is not detected (scale bar: 100 µm). **e** DAB immunostaining quantification in negative control, aged, and AD samples (*p < 0.05, ***p < 0.001 comparing to negative control, ^#^*p* < 0.05 comparing to aged, one-way ANOVA followed by a Bonferroni’s multiple comparisons post hoc test) (*n* = 4, 4, and 3, respectively). **f** Construct used to generate Tg(CaMKII-hA_2A_R) rats; Tg(CaMKII-hA_2A_R) animals present an overexpression of total A_2A_R in the hippocampus evaluated by in situ hybridization (both with the hA_2A_R probe and through cross-hybridization of the rat A_2A_R probe to the human A_2A_R mRNA). **g** Compositional images of fluorescence immunohistochemistry of hippocampus of WT and Tg(CaMKII-hA_2A_R) animals (scale bar: 1000 μm). Nuclei are labeled in blue (with Hoechst) and A_2A_R in red. A_2A_R staining is present in hippocampal areas of Tg(CaMKII-hA_2A_R) animals but not in WT littermates. Within the hippocampus, positive labeling can be observed in CA3 axonal projections and strong staining is also observed in the neuropil of DG and CA1 areas. On the top, z-stack maximum intensity projection images taken at 100 × magnification in CA1 area of hippocampus are presented (scale bar: 15 μm). MAP2 positive cells are identified by green fluorescence. A_2A_R staining can be observed in the neuropil of CA1 area in Tg(CaMKII-hA_2A_R) hippocampal slices. At the middle and bottom panels, a z-stack maximum intensity projection image taken at 100 × magnification in CA1 area of hippocampus is presented (scale bar: 15 μm). GFAP positive cells are identified by green fluorescence. No co-localization is found between A_2A_R and GFAP staining. **h** Immunoblotting analysis after subcellular fractionation of hippocampal tissue from WT and Tg(CaMKII-hA_2A_R) animals. **i** Electron micrographs of the area where the recordings were conducted in the hippocampus of Tg(CaMKII-hA_2A_R) animals showing immunogold particles for A_2A_R in the presynaptic neuron. On top, intracellular distribution of nanoparticles reveal a preferential presynaptic localization of A_2A_R. On the right, duplicates of the images with the identification of the subcellular structures. Pre presynaptic neuron, Post postsynaptic neuron, Mit mitochondria, SynV synaptic vesicle. Uncropped gels and blots with molecular weight standards are provided in Supplementary Fig. 6. All values are mean ± SEM

There is a genetic association of the adenosine A_2A_ receptor encoding gene (*ADORA2A*) with hippocampal volume in mild cognitive impairment and Alzheimer’s disease [19]. Plus, A_2A_R upregulation in cortex and hippocampus is associated with memory dysfunction in different animal models [45, 46]. We now probed this increase in human brain of aged and AD subjects. A_2A_R expression was measured in young (20–40 years old), aged (60–75 years old) and AD (60–75 years old, Braak stages 5–6) forebrain. There was a significant increase in A_2A_R protein levels in the aged forebrain that was further enhanced in samples from AD patients (Fig. 1a, b). The messenger RNA (mRNA) quantification by quantitative PCR (qPCR) indicates a 4.9 ± 0.3 (*n* = 3) fold increase in A_2A_R transcripts in AD samples compared to aged samples (Fig. 1c). To assess the cellular origin of this A_2A_R upregulation, we performed a histological analysis of the hippocampi from AD patients and age-matched controls. We detected a DAB-specific staining for A_2A_R in aging and AD sections (Fig. 1d, e), absent in the negative control (Supplementary Fig. 1a). In both conditions, we observed a neuron-specific A_2A_R positive staining (brown arrows; characterized by a large hypochromatic nucleus with nucleolar inclusions). We did not detect any significant A_2A_R signal in astrocytes (black arrows; nuclei typically have pale, finely granular chromatin patterns and relatively small or indistinct nucleoli), oligodendrocytes (blue arrows; small, round, relatively dark nuclei) or microglia (green arrows; rod-shaped and often irregularly contoured nuclei) [47] (Fig. 1d).

### Physiopathological levels of A_2A_R in neurons impair hippocampus-dependent spatial memory

Given that A_2A_R upregulation is associated with decreased cognitive performance characteristic of aging and AD, we studied a rat transgenic model with physiopathological levels of A_2A_R expression to address the underlying mechanism. These transgenic rats selectively overexpress the human A_2A_R in neurons under the control of the CaMKIIα promoter [Tg(CaMKII-hA_2A_R); Fig. 1f], mainly in the cortex and hippocampus, in an aging-like pattern of expression [23, 30]. The hippocampus displays a significant overexpression of A_2A_R, particularly the DG and CA1, as reported by the in situ A_2A_R mRNA human probe (Fig. 1f) and immunostaining (Fig. 1g) and negligible expression in other brain areas (see also [30]). Importantly, at 12–16 weeks of age, Tg(CaMKII-hA_2A_R) animals present a 5–8-fold increase of hippocampal A_2A_R immunoreactivity [30], which is of the same magnitude as the increase found in our human aged and AD samples (Fig. 1b), and equivalent to that of aged rats [22]. To further evaluate the profile of A_2A_R expression, co-staining for A_2A_R, GFAP, and MAP2 was performed in hippocampal slices, confirming the upsurge in the neuropil and discarding the possibility of astrocytic A_2A_R expression in this model (Fig. 1g). Biochemical fractionation of hippocampal tissue revealed a clear enrichment of A_2A_R in the SNAP25 positive fraction, in contrast to the low levels in the PSD95-enriched fraction, favoring a mainly presynaptic localization (Fig. 1h), as occurs for native A_2A_R in the rodent hippocampus [21, 48]. This was further confirmed by immunohistochemical analysis of the CA1 area of the hippocampus, in which the A_2A_R signal overlaps with that of SNAP25, and not with PSD95 signal (Supplementary Figs. 1b, c and 2a). This is not due to lack of resolution since our system was able to resolve a control section labeled for both a pre- and a postsynaptic protein (Supplementary Fig. 2b).

**Fig. 2:**
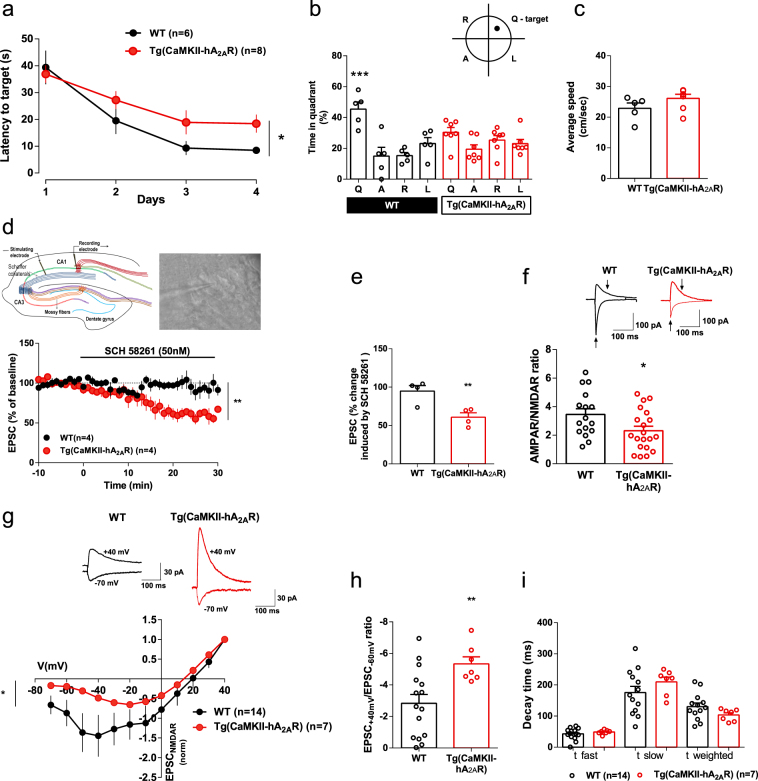
Physiopathological levels of A_2A_R in neurons impair hippocampus-dependent spatial memory and increase NMDAR currents in CA1 pyramidal neurons. **a**, **b** Hippocampal-dependent memory performance was assessed by the MWM test, in which acquisition (**a**) (*p < 0.05, two-way ANOVA) (*n* = 6 and 8, respectively) and retention (**b**) (***p < 0.001, one-way ANOVA followed by a Bonferroni’s multiple comparisons post hoc test within groups) (*n* = 6 and 8, respectively) were evaluated. **c** No changes in swimming speed during probe test between WT and Tg(CaMKII-hA_2A_R) animals (*n* = 6 and 8, respectively). **d** Top left, schematic representation of the simplified circuitry of the hippocampus. A stimulation electrode was placed in the Schaffer collaterals and the recording electrode patching a pyramidal cell of the CA1 area. Top right, pyramidal area of the CA1 with a recording electrode patching one cell. Graph: Averaged time course of excitatory postsynaptic currents after perfusion with SCH 58261 (50 nM) for 30 min, in neurons from WT and Tg(CaMKII-hA_2A_R) animals (**p < 0.01, unpaired *t*-test) (*n* = 4). Black traces represent baseline, while gray traces correspond to the EPSCs 20–30 min after SCH 58261 perfusion. **e** Averaged EPSCs (change in EPSCs from the last 10 min of SCH 58261 application) from acute SCH 58261 perfusion experiments. (**p < 0.01, unpaired *t*-test) (*n* = 4). **f** AMPAR/NMDAR ratio in neurons from WT and Tg(CaMKII-hA_2A_R) animals (*p < 0.05, unpaired *t*-test) (*n* = 16 and 20, respectively); representative traces of EPSCs recorded at −70 mV and + 40 mV, arrows indicate the amplitudes considered to calculate AMPAR/NMDAR ratio. **g** Plots of normalized EPSC_NMDA_ current–voltage relationships recorded in the presence of DNQX (100 µM) from WT and Tg(CaMKII-hA_2A_R) neurons (*p < 0.05, two-way ANOVA) (*n* = 14 and 7, respectively); representative traces of NMDAR EPSCs recorded at −70 mV and + 40 mV. **h** NMDAR EPSC_+40mV_/EPSC_-60mV_ ratio from WT and Tg(CaMKII-hA_2A_R) neurons (**p < 0.01, unpaired *t*-test) (*n* = 14 and 7, respectively). **i** Average time constants for fast and slow components (*τ*_fast_ and *τ*_slow_) of NMDAR EPSC recorded in neurons from WT and Tg(CaMKII-hA_2A_R) animals (*n* = 14 and 7, respectively). All values are mean ± SEM

Accordingly, immunoelectron micrographs of the CA1 area of Tg(CaMKII-hA_2A_R) reveal a preferential presynaptic localization of A_2A_R (Fig. 1i). We then evaluated hippocampus-dependent spatial memory using the Morris water maze (MWM) test. Transgenic animals displayed a decrease in acquisition (Fig. 2a) and a lack of preference for the target quadrant during the probe test (Fig. 2b). We did not find differences in swimming speed between groups (Fig. 2c).

### Increased levels of A_2A_R enhance glutamate release probability

To further dissect the mechanism by which A_2A_R impair memory performance, whole-cell patch-clamp recordings were performed. We first measured the intrinsic excitability of CA1 neurons from Tg(CaMKII-hA_2A_R) and WT rats. No changes were observed in passive properties (resting membrane potential or membrane resistance), nor in single spike analysis of the studied populations of neurons (Supplementary Fig. 3a, b). Moreover, neurons from WT and Tg(CaMKII-hA_2A_R) animals exhibited similar behavior when submitted to steps of current injection (Supplementary Fig. 3c–h). Thus, A_2A_R overexpression does not impact on the studied passive or intrinsic excitability properties of CA1 neurons. We then performed afferent-evoked EPSCs from CA1 pyramidal neurons (*V*_h_ = −70 mV), in the presence of GABA_A_ receptor antagonist picrotoxin (50 µM). A_2A_R blockade significantly inhibited excitatory postsynaptic currents (EPSCs), an effect that was not observed in WT animals (Fig. 2d, e). Thus, there is a gain of function of A_2A_R upon their overexpression, whereby A_2A_R tonically control basal synaptic transmission in Tg(CaMKII-hA_2A_R) animals, which does not occur in WT animals.

To test if tonic A_2A_R modulation of neuronal function occurs at a presynaptic level, we evaluated the glutamate release probability. A facilitation of the paired-pulse ratio (PPR) was observed in neurons from WT animals at all inter-stimulation intervals, more evident for the shorter intervals (Supplementary Fig. 3i). The magnitude of facilitation was reduced in Tg(CaMKII-hA_2A_R) rats when compared to WT neurons (Supplementary Fig. 3i), albeit maintaining the same facilitatory profile. These data suggest that neuronal A_2A_R overexpression increases glutamate release probability [49, 50]. These PPR alterations in Tg(CaMKII-hA_2A_R) rats were completely rescued by the A_2A_R selective antagonist, SCH 58261 (Supplementary Fig. 3i). As expected, A_2A_R blockade does not alter PPR values in WT animals (Supplementary Fig. 3i).

### A_2A_R increase NMDAR-mediated currents in CA1 pyramidal neurons

A_2A_R were proposed to mainly modulate NMDA receptors (NMDAR) [51–53], which are minor contributors to excitatory synaptic transmission under basal conditions in the hippocampus [54]. We tested possible alterations of the AMPA and NMDA receptor contribution, by quantifying the AMPA/NMDA receptor ratio. The AMPA/NMDA receptor ratio was decreased in Tg(CaMKII-hA_2A_R) vs. WT animals (Fig. 2f). To assess if this could be attributed to changes in the gating properties of the receptors, we performed current–voltage (I–V) relationships in pharmacologically isolated AMPAR and NMDAR responses. While the I–V relationships of the NMDAR were significantly increased in neurons from Tg(CaMKII-hA_2A_R) animals (Fig. 2g), the AMPAR voltage-dependency was unaltered in Tg(CaMKII-hA_2A_R) neurons (Supplementary Fig. 3j). Moreover, we calculated the ratio between NMDAR current recorded at + 40 mV and at −60 mV, and observed it was significantly increased in Tg(CaMKII-hA_2A_R) neurons (Fig. 2h).

In the hippocampus, NMDARs are heteromeric assemblies mainly composed of a constitutive GluN1 subunit and GluN2A or GluN2B subunits [55]. The deactivation time course of GluN1/GluN2B heteromers is higher than the one observed for GluN1/GluN2A heteromers [56]. To test if NMDAR overactivation was due to alterations in NMDAR subunit composition, we analyzed the deactivation kinetics of pharmacologically isolated NMDAR EPSCs. Time constants for fast, slow and weighted components (*τ*_fast_, *τ*_slow_, and τ_weighted_) were obtained by fitting the pharmacologically isolated NMDAR EPSCs (*V*_h_ = + 40 mV) to a double exponential function (Levenberg-Marquandt method). No differences were found between groups for all parameters evaluated (Fig. 2i), suggesting that the enhancement of NMDAR conductance observed in Tg(CaMKII-hA_2A_R) neurons is not related to alterations in NMDAR subunit composition.

### Physiopathological levels of A_2A_R lead to a NMDAR-mediated LTD-to-LTP shift

In view of the key role of NMDAR in the control of synaptic plasticity we next focused on the impact of A_2A_R overexpression on long-term depression (LTD) in the CA1 area of the dorsal hippocampus. LTD is altered in association with memory deficits in aging [57] and animal models of stress [58] or AD [59]. In the hippocampus, LTD can be experimentally induced using several different protocols, including both electrical and pharmacological stimulation [60]. For our purpose, we selected a low-frequency stimulation (LFS) protocol particularly efficient in inducing robust LTDs in adult animals—three trains of 1200 pulses, 2 Hz, 10-min interval [39, 61].

**Fig. 3:**
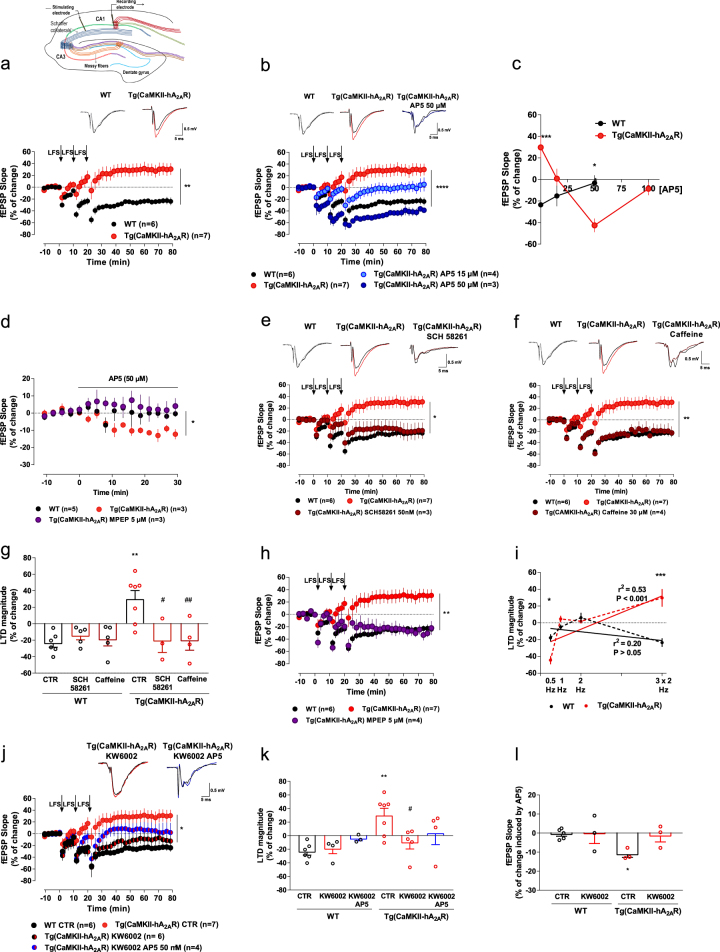
Physiopathological levels of A_2A_R lead to a NMDAR-mediated LTD-to-LTP shift. **a** Top, schematic representation of the simplified circuitry of the hippocampus. A stimulation electrode was placed in the Schaffer collaterals and the recording electrode in the pyramidal layer of the CA1 area. Graph: Changes in fEPSP slope induced by LFS stimulation (three trains of 1200 pulses, 2 Hz) recorded from WT and Tg(CaMKII-hA_2A_R) hippocampal slices (**p < 0.01, unpaired *t*-test) (*n* = 6 and 7, respectively); representative traces of fEPSPs before (black) and 50–60 min after (gray, red) LTD induction in WT and Tg(CaMKII-hA_2A_R) animals. **b** Changes in fEPSP slope induced by LFS stimulation (three trains of 1200 pulses, 2 Hz) recorded from WT and Tg(CaMKII-hA_2A_R) hippocampal slices after partial and complete NMDAR blockade with AP5 (15 and 50 µM, respectively) (****p < 0.0001 comparing to Tg(CaMKII-hA_2A_R), two-way ANOVA followed by Bonferroni’s multiple comparisons post hoc test) (*n* = 4 and 3, respectively); representative traces of fEPSPs before (black) and 50–60 min after (gray, red, blue) LTD induction in WT, Tg(CaMKII-hA_2A_R) and Tg(CaMKII-hA_2A_R) animals with NMDAR complete blockade. **c** Effect of increasing AP5 concentrations (0–100 µM) on synaptic strength after low-frequency stimulation (changes after 50-60 min) in WT and Tg(CaMKII-hA_2A_R) animals (*p < 0.05, ***p < 0.001, two-way ANOVA followed by Bonferroni’s multiple comparisons post hoc test) (WT: *n* = 6, 4, and 4, respectively; Tg(CaMKII-hA_2A_R): *n* = 7, 4, 3, and 3, respectively). **d** mGluR5 blockade rescues the effect of AP5 on basal transmission in Tg(CaMKII-hA_2A_R) animals (*p < 0.05 comparing to WT, one-way ANOVA followed by Bonferroni’s multiple comparisons post hoc test) (*n* = 3). **e** Changes in fEPSP slope induced by LFS stimulation recorded from WT, Tg(CaMKII-hA_2A_R) and Tg(CaMKII-hA_2A_R) hippocampal slices perfused with SCH 58261 (50 nM) (*p < 0.05 comparing to Tg(CaMKII-hA_2A_R), two-way ANOVA followed by Bonferroni’s multiple comparisons post hoc test) (*n* = 6, 7, and 3, respectively); representative traces of fEPSPs before (black) and 50–60 min after (gray, red, dark red) LTD induction in WT, Tg(CaMKII-hA_2A_R) and Tg(CaMKII-hA_2A_R) animals with SCH 58261. **f** Changes in fEPSP slope induced by LFS stimulation recorded from WT, Tg(CaMKII-hA_2A_R) and Tg(CaMKII-hA_2A_R) hippocampal slices perfused with caffeine (30 µM) (**p < 0.01 comparing to Tg(CaMKII-hA_2A_R), two-way ANOVA followed by Bonferroni’s multiple comparisons post hoc test) (*n* = 6, 7, and 4, respectively); representative traces of fEPSPs before (black) and 50–60 min after (gray, red, dark red) LTD induction in WT, Tg(CaMKII-hA_2A_R) and Tg(CaMKII-hA_2A_R) animals with caffeine. **g** Changes in fEPSP slope induced by LFS stimulation recorded from WT and Tg(CaMKII-hA_2A_R) perfused with SCH 58261 (50 Mn) or caffeine (30 µM) (**p < 0.01 comparing to WT, ^#^*p* < 0.05, ^##^*p* < 0.01 comparing to Tg(CaMKII-hA_2A_R), two-way ANOVA followed by Bonferroni’s multiple comparisons post hoc test) (WT: *n* = 5). **h** Changes in fEPSP slope induced by LFS stimulation recorded from WT, Tg(CaMKII-hA_2A_R) and Tg(CaMKII-hA_2A_R) hippocampal slices perfused with mGluR5 antagonist MPEP (5 µM) (**p < 0.01 comparing to Tg(CaMKII-hA_2A_R), two-way ANOVA followed by Bonferroni’s multiple comparisons post hoc test) (*n* = 6, 7, and 4, respectively). **i** Changes in fEPSP slope induced by increasing frequencies of LFS stimulation in WT and Tg(CaMKII-hA_2A_R) animals (*p < 0.05, ***p < 0.001, two-way ANOVA followed by Bonferroni’s multiple comparisons post hoc test) (WT: *n* = 4, 3, 3, and 6, respectively; Tg(CaMKII-hA_2A_R): 4, 4, 4, and 7, respectively). **j** Changes in fEPSP slope induced by LFS stimulation (three trains of 1200 pulses, 2 Hz) in WT and Tg(CaMKII-hA_2A_R) animals non-treated (CTR) and treated with KW6002 (**p < 0.01 comparing to Tg(CaMKII-hA_2A_R, two-way ANOVA followed by Bonferroni’s multiple comparisons post hoc test) (*n* = 6). AP5, 50 µM, abolished LTD in Tg(CaMKII-hA_2A_R) animals treated with KW6002 (*n* = 4); representative traces of fEPSPs before (black) and 50–60 min after (red, blue) LTD induction in Tg(CaMKII-hA_2A_R) chronically treated with KW6002 in the absence and presence of AP5. **k** Changes in fEPSP slope induced by LFS stimulation recorded from WT and Tg(CaMKII-hA_2A_R) animals non-treated (CTR) and treated with KW6002, in the presence and absence of AP5 (50 µM). (**p < 0.01 comparing to WT, ^#^*p* < 0.05 comparing to Tg(CaMKII-hA_2A_R), two-way ANOVA followed by Bonferroni’s multiple comparisons post hoc test) (WT: *n* = 6, 4, and 3, respectively; Tg(CaMKII-hA_2A_R): *n* = 7, 5, and 4, respectively). **l** Chronic KW6002 treatment reverts the effect of AP5 on basal transmission in Tg(CaMKII-hA_2A_R) animals (*p < 0.05 comparing to WT, one-way ANOVA followed by Bonferroni’s multiple comparisons post hoc test) (*n* = 3). All values are mean ± SEM

We observed a significant alteration of the pattern of induction of LTD: whereas this protocol triggered a typical LTD in WT animals, it generated instead a significant LTP in Tg(CaMKII-hA_2A_R) animals (Fig. 3a).

The pattern of activation of NMDAR controls the entry of calcium into the postsynaptic compartment, determining the output of plasticity [14, 62]. The robust recruitment of NMDAR causes a large calcium influx driving LTP, whereas the engagement of a lower number of NMDAR causes a more discrete calcium influx culminating in LTD [14, 62]. To confirm a greater NMDAR role in this LTD-to-LTP shift in Tg(CaMKII-hA_2A_R) animals, we induced LTD and titrated the recruitment of NMDAR using increasing concentrations of the NMDAR antagonist, AP5 (Fig. 3b, c). With a low concentration of AP5 (15 µM), the LTP observed in Tg(CaMKII-hA_2A_R) animals was abolished. Further increase of the AP5 concentration to 50 µM converted the LTP into LTD, fully rescuing the abnormal plasticity profile in Tg(CaMKII-hA_2A_R) to a WT-like phenotype. Further increase of AP5 concentration to a supra-maximal value of 100 µM abolished LTD, confirming that LTD in Tg(CaMKII-hA_2A_R) animals is still strictly NMDAR-dependent (Fig. 3c). In WT animals, LTD magnitude did not change with 15 µM of AP5 (Fig. 3c), but when NMDAR were blocked with AP5 at 50 µM, no LTD was elicited, as expected (Fig. 3c). Consistent with an aberrant NMDAR contribution to basal transmission in Tg(CaMKII-hA_2A_R) animals, we observed a decrease in fEPSPs slope with AP5 (50 µM) in Tg(CaMKII-hA_2A_R) animals, but not in WT animals (Fig. 3d). Acute blockade of A_2A_R directly on slices rescued the LTD shift observed in Tg(CaMKII-hA_2A_R) animals. In fact, LFS stimulation of Tg(CaMKII-hA_2A_R) slices with either SCH 58261 (50 nM) or the non-selective adenosine antagonist, caffeine (30 µM) triggered an LTD similar to that found in WT animals (Fig. 3e–g). As expected, in WT animals, this A_2A_R blockade did not change LTD magnitude (Fig. 3g). Accordingly, SCH 58261, 50 nM, significantly decreased basal field excitatory post-synaptic potentials (fEPSPs) in Tg(CaMKII-hA_2A_R) animals, while no effect was observed in WT (Supplementary Fig. 3k), confirming that the effects seen in Tg(CaMKII-hA_2A_R) animals are indeed due to A_2A_R overactivation.

Group I metabotropic glutamate receptors, namely mGluR5, are postsynaptic and tightly coupled to NMDA receptors [51, 63, 64], conferring them the ability to exacerbate NMDAR-mediated toxicity. Upon activation by glutamate release, preferentially upon strong synaptic activation, they increase NMDAR-mediated Ca^2+^ currents [65]. When we blocked mGluR5 with MPEP, 5 µM, the LTD-to-LTP shift observed in Tg(CaMKII-hA_2A_R) animals was prevented (Fig. 3h). Consistent with their activation upstream of NMDAR, the aberrant NMDAR component in Tg(CaMKII-hA_2A_R) disappeared upon mGluR5 blockade (Fig. 3d), disclosing mGluR5 as a player in the observed A_2A_R-induced synaptic dysfunction. MPEP does not change AP5-induced basal transmission or LTD magnitude in WT animals (Supplementary Fig. 3l, m).

To further study the alterations in the threshold for LTD in Tg(CaMKII-hA_2A_R) animals, we elicited LTD using decreasing frequencies of stimulation maintaining the total number of pulses of one train (1200): 2, 1, and 0.5 Hz. In contrast to what we observed for 3 × trains of 1200 pulses (2 Hz), a lower 0.5 Hz frequency was more effective in inducing LTD in Tg(CaMKII-hA_2A_R) than in WT animals (Fig. 3i and Supplementary Fig. 4a). Furthermore, frequencies of 1 and 2 Hz failed to elicit LTD in both WT and Tg(CaMKII-hA_2A_R) (Supplementary Fig. 4b, c). More importantly, the magnitude of LTD in Tg(CaMKII-hA_2A_R) animals correlated significantly with the frequency of stimulation (Fig. 3i), consistent with a shift to the left in the LTD threshold.

### Blockade of A_2A_R activation in vivo rescues the LTD-to-LTP shift in Tg(CaMKII-hA_2A_R) animals

To establish that A_2A_R overactivation is indeed the trigger for the aberrant NMDAR recruitment, we treated Tg(CaMKII-hA_2A_R) animals with the A_2A_R selective antagonist KW6002 (5 mg/kg/day), in the drinking water for 4 weeks. In Tg(CaMKII-hA_2A_R)-treated animals, LFS induced an LTD comparable to WT animals, rescuing the LTD-to-LTP shift (Fig. 3j, k). Furthermore, the KW6002 treatment normalized NMDAR overactivation, as confirmed by the reinstatement of AP5 ability to fully block LTD in Tg(CaMKII-hA_2A_R) (Fig. 3j, k). The treatment with KW6002 did not change LTD magnitude in WT animals (Fig. 3k), nor A_2A_R mRNA relative expression in both WT and Tg(CaMKII-hA_2A_R) (Supplementary Fig. 4d). The increased NMDAR contribution to basal transmission observed in Tg(CaMKII-hA_2A_R) animals disappeared upon chronic KW6002 treatment (Fig. 3l).

### Increased levels of A_2A_R impair calcium homeostasis

**Fig. 4:**
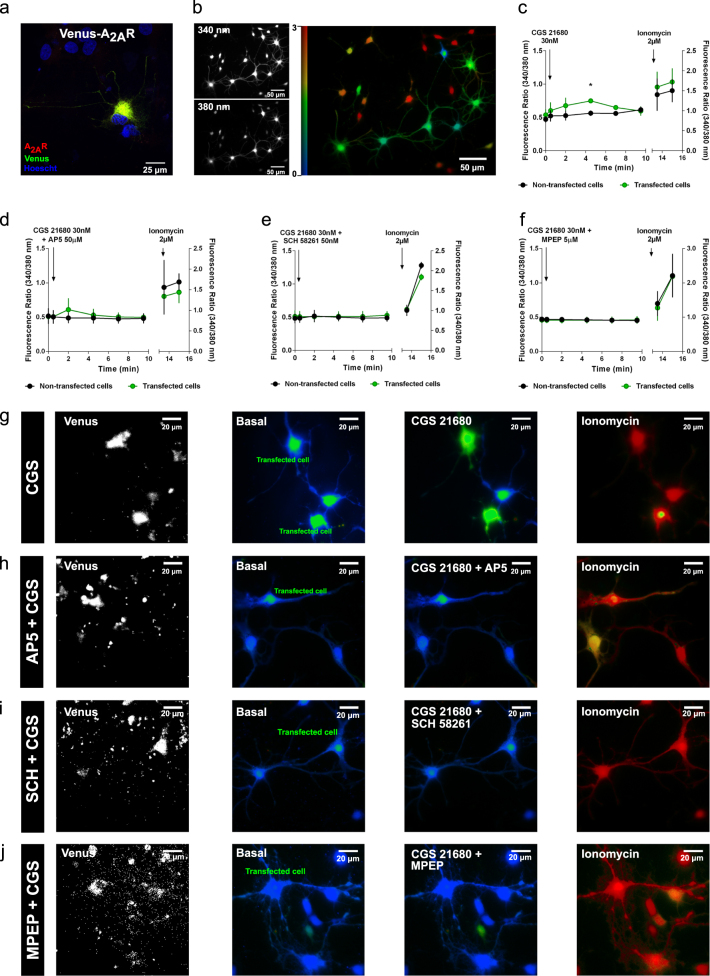
Increased levels of A_2A_R impair calcium homeostasis. **a** Control immunocytochemistry analysis of neurons transfected with Venus-A_2A_R construct confirmed co-expression of Venus and A_2A_R. **b** Representative images of Ca^2+^ imaging. Bright regions indicate the location of cytoplasm and organelles, where the concentration of Ca^2+^ is higher than in the dark regions indicating the extracellular medium, where diffusion processes take place. The right image corresponds to the ratio between the radiation emitted at 510 nm, when cells are excited at 340 nm, over emission upon excitation at 380 nm (F340/F380). **c** Time course of Ca^2+^-dependent fluorescence recorded and averaged per minute from Fura-2 AM neurons transfected with Venus-A_2A_R construct in response to CGS 21680, 30 nM, and ionomycin, 2 µM. Application of A_2A_R agonist CGS 21680, 30 nM, elevated intracellular Ca^2+^ levels in Venus-A_2A_R transfected neurons, whereas lower changes in fluorescence were detected in non-transfected neurons (*p < 0.05, unpaired *t*-test). Time of application of drugs are shown by arrows. (4–15 responsive cells per experimental condition from three independent cultures). **d** Time course of Ca^2+^-dependent fluorescence recorded and averaged from Fura-2 AM neurons transfected with Venus-A_2A_R construct in response to AP5, 50 µM, CGS 21680, 30 nM, and ionomycin, 2 µM. The A_2A_R-evoked increase in [Ca^2+^]i observed in **c**) was prevented by NMDAR antagonism (4–15 responsive cells per experimental condition from two independent cultures). Time of application of drugs are shown by arrows. **e** Time course of Ca^2+^-dependent fluorescence recorded and averaged from Fura-2 AM neurons transfected with Venus-A_2A_R construct in response to SCH 58261, 50 nM, CGS 21680, 30 nM, and ionomycin, 2 µM. The A_2A_R-evoked increase in [Ca^2+^]i observed in **c**) was prevented by A_2A_R antagonism (4–15 responsive cells per experimental condition from two independent cultures). Time of application of drugs are shown by arrows. **f** Time course of Ca^2+^-dependent fluorescence recorded and averaged from Fura-2 AM neurons transfected with Venus-A_2A_R construct in response to MPEP, 5 µM, CGS 21680, 30 nM, and ionomycin, 2 µM. The A_2A_R-evoked increase in [Ca^2+^]i observed in **c**) was prevented by mGluR5 antagonism (4–15 responsive cells per experimental condition from three independent cultures). Time of application of drugs are shown by arrows. **g**, **h**, **i**, **j** Representative images of the different conditions showed in (**c**, **d**, **e**), and **f**, respectively. All values are mean ± SEM

To investigate whether A_2A_R-mediated NMDAR hyperactivation disrupted Ca^2+^ signaling, we measured variations in intracellular calcium concentrations ([Ca^2+^]i) in primary neuronal cultures transfected with A_2A_R. For this, we used a construct encoding a Venus-A_2A_R fusion protein. We confirmed the co-localization of the Venus signal with the immunostaining for A_2A_R (Fig. 4a). Changes in [Ca^2+^]i were detected by Ca^2+^ imaging using Fura 2-acetoxymethyl ester (Fura-2 AM) (Fig. 4b). Application of the A_2A_R agonist CGS 21680, 30 nM, elevated intracellular Ca^2+^ levels in Venus-A_2A_R transfected neurons, whereas in non-transfected neurons lower changes in fluorescence were detected (Fig. 4c, g and Supplementary Video 1). This A_2A_R-evoked increase in [Ca^2+^]i was prevented by the NMDAR antagonist, AP5, 50 μM (Fig. 4d, h), the A_2A_R antagonist, SCH 58261, 50 nM (Fig. 4e, i) or mGluR5 antagonist MPEP, 5 μM (Fig. 4f, j). These results show for the first time a crosstalk between A_2A_R and NMDAR that impacts on Ca^2+^ influx in glutamatergic neurons.

### LTD-to-LTP shift in aged and APP/PS1 animals is rescued by A_2A_R blockade

Aging and AD are associated with an upregulation of A_2A_R in the hippocampus as we report here (Fig. 1) and others have shown previously [20, 21, 23, 66]. We evaluated putative LTD impairments in aged animals and in an APP/PS1 mouse model of AD, both models displaying A_2A_R increased levels (Supplementary Fig. 4e, f).

**Fig. 5:**
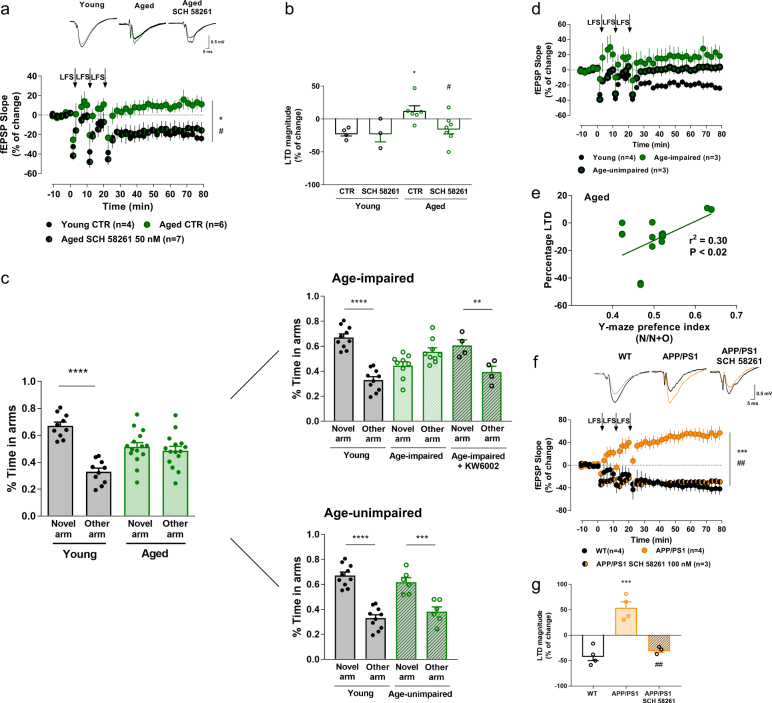
LTD-to-LTP shift in aged and APP/PS1 animals is rescued by A_2A_R blockade. **a**, **b** Changes in fEPSP slope induced by LFS stimulation recorded from young and aged animals upon acute A_2A_R blockade (SCH 58261, 50 nM) (*p < 0.05 comparing to young, ^#^*p* < 0.05 comparing to aged, two-way ANOVA followed by Tukey’s multiple comparisons post hoc test) (Young: 3 and 4, respectively; Aged: 6 and 7, respectively); representative traces of fEPSPs before (black) and 50–60 min after (gray, green, dark green) LTD induction in young, aged, and aged animais treated with SCH 58261, respectively. **c** Spatial memory performance was assessed by the Y-Maze test. Aged animals displayed a loss of preference for the novel arm (***p < 0.001, ****p < 0.0001, novel arm comparing to other arm, two-way ANOVA followed by Bonferroni’s multiple comparisons post hoc test) (young: *n* = 10; aged: *n* = 15). The pool of aged rats included a substantial subset of rats that performed within the range of young rats, labeled age-unimpaired rats (*n* = 6), while other clearly showed impairment, labeled age-impaired rats (*n* = 9), that is rescued upon chronic KW6002 treatment (**p < 0.01, novel arm comparing to other arm, two-way ANOVA followed by Bonferroni’s multiple comparisons post hoc test) (*n* = 4). **d** Age-impaired animals exhibited a LTD-to-LTP shift (*p < 0.05 comparing to WT, one-way ANOVA followed by a Bonferroni’s multiple comparisons post hoc test) (*n* = 3). Age-unimpaired animals can be distinguished by their lack of response to LFS (*n* = 3). **e** LTD magnitude observed in aged animals significantly correlates with Y-maze preference index (three replicates of *n* = 6 animals, *r*^2^ = 0.30, *p* < 0.05). **f**, **g** Changes in fEPSP slope induced by LFS stimulation recorded from WT, APP/PS1 mice, and APP/PS1 hippocampal slices perfused with SCH 58261 (100 nM) (***p < 0.001 comparing to WT, ^##^*p* < 0.01 comparing to APP/PS1, one-way ANOVA followed by a Bonferroni’s multiple comparisons post hoc test) (*n* = 4, 4, and 3, respectively); representative traces of fEPSPs before (black) and 50–60 min after (gray, orange) LTD induction in WT, APP/PS1 and APP/PS1 SCH 58261 100 nM. All values are mean ± SEM

Aged animals displayed the same LTD-to-LTP shift to that observed in our Tg(CaMKII-hA_2A_R) animals, while in young animals a robust LTD was achieved (Fig. 5a, b). The LTD-to-LTP shift was completely rescued with A_2A_R blockade by SCH 58261 (Fig. 5a, b), whereas SCH 58261 did not alter LTD profile in young animals (Fig. 5b). Within the aged group, we identified a subset of age-impaired animals that performed worse than young rats in the Y-maze test, revealing no preference for the novel arm (Fig. 5c). Interestingly, these same animals seem to be distinguished by an LTD-to-LTP shift, also observed in Tg(CaMKII-hA_2A_R) (Fig. 5d). In contrast, age-unimpaired animals performed within the range of young rats (Fig. 5c) and could be distinguished their lack of response to LFS (Fig. 5d). Consistent with the enhanced role of A_2A_R upon aging [23, 25, 28], SCH 58261 decreased basal transmission in hippocampal slices of aged animals, while no effect was observed in young animals (Supplementary Fig. 4g). We observed a tendency towards an increased effect of SCH 58261 in age-impaired subset (Supplementary Fig. 4h), when compared with age-unimpaired animals, in spite of the lower *n*. This larger tonic effect of adenosine suggests an increased A_2A_R activation in age-impaired animals. Importantly, we found that plasticity profile correlated significantly with the behavioral memory index in aged rats (Fig. 5e), whereby a higher LTD-to-LTP shift corresponded to a worse Y-maze performance. Notably, a 3-week treatment with the selective A_2A_R antagonist (KW6002; 5 mg/Kg/day; oral) restored memory impairments, as observed by the increased time spent in the novel arm (Fig. 5c). This KW6002 treatment did not affect A_2A_R mRNA expression in aged animals (Supplementary Fig. 4e), consistent to what was observed for Tg(CAMKII-hA_2A_R) animals (Supplementary Fig. 4d).

In 11–12 months old APP/PS1 mice, these animals display memory deficits [67] and LFS elicited LTP instead of LTD (Fig. 5f, g), as seen in Tg(CaMKII-hA_2A_R) animals. Importantly, acute blockade of A_2A_R with 100 nM SCH 58261, was able to revert the LTD-to-LTP shift back to the LTD characteristic of WT mice (Fig. 5f, g).

## Discussion

We show that the A_2A_R upsurge, described in different pathological situations in rodent models, such as hypoxia, ischemia, stress, diabetes, and even upon aging [44], is also characteristic of the human aged brain and is aggravated in AD [68]. Moreover, we describe that an increase in neuronal A_2A_R is sufficient to drive deficits in synaptic plasticity, leading to an LTD-to-LTP shift and impairments of hippocampal-dependent learning and memory. This is a consequence of an A_2A_R-induced increase in postsynaptic Ca^2+^ influx via NMDAR, which is dependent on mGluR5 activation (see Supplementary Fig. 5 for a summary). We reveal that the same synaptic plasticity shift occurs in the hippocampus of aged and APP/PS1 animals, which is rescued upon A_2A_R blockade.

The findings in human hippocampal samples confirm the observations made in animal models, in which A_2A_R density is increased upon aging [20, 22–24, 69]. Accordingly, in humans, several epidemiological studies have shown that regular caffeine consumption attenuates memory disruption during aging and decreases the risk of developing memory impairments in AD patients [34, 40–43]. Furthermore, in animal models of several other pathologies, there is a clear correlation of hippocampal A_2A_R upregulation with cognitive deficits, such as in acute or chronic stress [38, 45, 70], Alzheimer’s [26], Parkinson’s [71], or Huntington’s diseases [72, 73]. However, the exact mechanism by which neuronal A_2A_R overactivation could trigger or increase the susceptibility for memory dysfunction in these multiple pathologies was not known.

A previous study using a model of A_2A_R overexpression under the control of the neuron-specific enolase promoter reported working memory deficits [74]. However, that study could not distinguish A_2A_R-related embryonic effects from those elicited by postnatal alterations. In contrast, our model of overexpression, driven by a CaMKII promoter, allows a progressive postnatal and forebrain-specific expression, bypassing developmental effects and closer to an age-like A_2A_R distribution. These animals exhibit depressive-like behavior, hyperlocomotion, and altered exploratory behavior, consistent with the depressive signs found in aging, chronic stress, and AD [75]. Importantly, they do not present changes in adenosine A_1_ receptor levels nor adenosine levels in the hippocampus [30]. Furthermore, at 12 week-old, the Tg(CaMKII-hA_2A_R) animals display a 5–8-fold increase of A_2A_R immunoreactivity [30], which is in the same magnitude of that found in our human aged and AD samples, and equivalent to that of aged rats [20–22, 25]. Importantly, this A_2A_R overexpression occurs in the hippocampus and cortex, recapitulating the pattern observed in our aged and AD human samples and consistent with previous reports [68]. In the Tg(CaMKII-hA_2A_R) model there is no evidence of A_2A_R overexpression in astrocytes, strengthening the idea that the observed memory and synaptic impairments are due to a neuronal-specific A_2A_R overexpression. The fact that in aged and AD human samples we observed a clear A_2A_R overexpression in neurons further emphasizes neuronal A_2A_R as key mediators in synaptic glutamatergic dysfunction observed in aging and AD [76]. Aberrant astrocytic A_2A_R expression in late-stage AD has been associated to cognitive decline, and indeed astrocytic A_2A_R can lead to alterations of synaptic A_2A_R-mediated functions [77]. However, neuronal contribution is highlighted by recent evidence showing that stimulation of neuronal opto-A_2A_R in the hippocampus induces changes in synaptic plasticity and CREB activation [27]. Moreover, silencing A_2A_R in neurons of the associative/commissural pathway rescues the aberrant LTP in APP/PS1 mice in a non-NMDAR-dependent mechanism [26, 27]. Our findings demonstrate that neuronal A_2A_ overactivation is sufficient to induce synaptic dysfunction and cognitive impairments. This suggests that synaptic dysfunction in aging and early stages of AD may be driven predominantly by a neuronal A_2A_R progressive dysfunction, whereas at later Braak stages of AD, astrocytic A_2A_R and inflammation might become more relevant [39, 76].

Both aging and AD comprehend functional and structural alterations in the hippocampus that drive cognitive decline [1, 78]. Furthermore, they are also characterized by an abnormal Ca^2+^ signaling. Several studies reported an age-associated increase in basal [Ca^2+^]i levels [79, 80] and action potential-evoked calcium influx [81] and a reduction in the expression of calcium-buffering proteins. In AD mouse models, increased levels of intracellular Ca^2+^ [82] distorts the normal Ca^2+^ signaling and Ca^2+^-dependent mechanisms and can indeed trigger the amyloidogenic pathway [83–85]. Concretely, the AD brain is characterized by a clear loss of synaptic processes and neuronal cell bodies in the limbic and association cortices (reviewed in ref. [1]). In normal aging, there is still a considerable structural preservation in several brain areas including the hippocampus [78, 86–88]. Therefore it is conceivable to hypothesize that the shift from normal aging to AD could be related to dysregulation of the integrated homeostatic network caused by differences either in the levels of the endogenous ligand —adenosine [89], or in the expression of A_2A_R that are increased upon aging and are further exacerbated in AD (Fig. 1). To specifically check the endogenous activation of A_2A_R, we have quantified the effect of blocking A_2A_R in CA1 basal transmission, in age-impaired and age-unimpaired animals. The fact that we observe a tendency towards an increased effect of SCH 58261 in age-impaired subset, without significant differences in the bulk mRNA A_2A_R levels within the aged group, supports the first hypothesis.

A_2A_R and A_1_R form heteromers and, under physiological conditions, adenosine preferentially activate A_1_R [90, 91] in the hippocampus, which control glutamatergic neurotransmission, namely by a decrease in NMDAR-mediated responses [92, 93]. In conditions where hippocampal transmission is dysfunctional, there is an upregulation of A_2A_R (reviewed in ref. [44]) together with an increased release of ATP as a danger signal [94], which is the main source of the extracellular adenosine activating A_2A_R [95]. The signaling of these upregulated A_2A_R is shifted from a PKC-dependent, controlled by inhibitory adenosine A_1_ receptors, towards a more disinhibited PKA-dependent mechanism in aging and pathology [25, 30, 96], leading to impaired synaptic plasticity and compromised memory performance [26, 38, 39, 45]

This dysfunction is associated to an excitatory effect on glutamatergic transmission, which we can postulate that it may be mediated by non-heteromerized A_2A_R. Our results in Tg(CaMKII-hA_2A_R) are in line with this hypothesis, since we observe an aberrant constitutive activation of A_2A_R, dependent on PKA [30], and consequent NMDAR, both contributing to basal synaptic transmission, that could not be observed in WT animals.

Long-term synaptic plasticity processes (LTP and LTD) are the main neurophysiological correlate of memory [7, 97]. Although the relation between hippocampal LTP and memory is the most explored [98], there is also robust evidence that altered hippocampal LTD affects memory performance [12, 99], as shown in animal models of stress [58] or of AD [59, 61]. We now report that Tg(CaMKII-hA_2A_R) animals display memory impairments together with a newly described LTD-to-LTP shift as a result of an increase in Ca^2+^ influx dependent on NMDAR activation. In fact, we observed a dose-dependent rescue of LTD in slices from Tg(CaMKII-hA_2A_R) rats with the NMDAR antagonist AP5. The concentration that fully restored LTD in Tg(CaMKII-hA_2A_R) prevented it in WT animals. This LTD in Tg(CaMKII-hA_2A_R) is NMDAR-dependent, since a higher concentration of AP5, 100 µM, was able to completely abolish LTD. Accordingly, in primary cultures of hippocampal neurons, A_2A_R activation directly increased Ca^2+^ intracellular levels through NMDAR activation, blocked by its selective antagonist AP5. These data strongly indicate an A_2A_R–NMDAR interaction, consistent with our synaptic plasticity results. Although in our paper we observe a Ca^2+^ influx-dependent LTD-to-LTP shift, there are reports that metabotropic NMDAR activity, independent of calcium influx, can also induce LTD [100]. More relevant, we have shown that the blockade of A_2A_R can restore a similar LTD-to-LTP shift in aged and AD mice models, strongly emphasizing A_2A_R as the pathophysiological mediator involved in this synaptic shift.

We can postulate that NMDA receptor gating properties are directly modulated by such an increase in glutamate available to activate the ionotropic receptor. In such a case, however, AMPA-mediated currents in Tg(CaMKII-hA_2A_R) would be similarly increased, which we do not find. Moreover, when we transfected neurons with A_2A_R we could only observe an increase in Ca^2+^ transients in transfected cells, but not in the adjacent non-transfected neurons. If an overall increase in glutamate were the only mechanism, then we might expect some non-transfected neurons to be affected. Therefore, other postsynaptic modifications due to an A_2A_R-related increase in glutamate release and/or postsynaptic A_2A_R contribution must be considered. Indeed, a postsynaptic activation of A_2A_R can lead to downstream activation of CREB in the hippocampus [27], and A_2A_R and mGluR5 can directly interact and regulate NMDAR activity [53, 101].

Group I metabotropic glutamate receptors, namely mGluR5, are postsynaptic and tightly coupled to NMDA receptors [63, 64, 102], conferring them the ability to either protect or exacerbate NMDAR-mediated toxicity depending upon the model or cell type [103]. Upon activation by glutamate release, preferentially upon strong synaptic activation, mGluR5 increase NMDAR-mediated Ca^2+^ currents [65], by reducing the Mg^2+^ block [103] and triggering the phosphorylation of NMDAR [64]. Previous studies hinted at a possible A_2A_R-NMDAR crosstalk, since A_2A_R can control expression [51, 102], recruitment [52] and the rate of desensitization [53] of NMDAR. We and others have provided compelling evidence of an A_2A_R-mGluR5 synergistic interaction in the modulation of NMDAR-mediated effects [53, 101, 102, 104]. Thus, mGluR5 is a likely candidate to act as a switch between A_2A_R and NMDAR, by sensing glutamate and translating it into NMDAR overactivation. Consistent with this hypothesis, we observe that mGluR5 blockade prevents the downstream NMDAR aberrant contribution in basal transmission and the LTD-to-LTP shift, supporting the mGluR5-NMDAR interplay as key player in the observed A_2A_R-induced physiopathology.

Aging is associated with a decline in cognitive function that can, in part, be explained by changes in the mechanisms of plasticity [78]. While some studies report increased susceptibility to LTD during aging [15], others do not observe alterations in LTD magnitude between young and aged animals [16]. These discrepancies can be easily explained by differences in rat strain, stimulation pattern and Ca^2+^/Mg^2+^ ratio. In fact, the stimulation pattern used in those studies (900 pulses, 1 Hz) does not elicit LTD in young animals [15, 105], while we and others observe a robust LTD with our LFS protocol [39, 61]. Moreover, those age differences were reverted under elevated Ca^2+^/Mg^2+^ ratio suggesting that aging is associated with a shift in the threshold for LTD-induction rather than in the LTD intrinsic capacity [16, 57]. Notably, the significant correlation between LTD magnitude and the frequency of LFS in Tg(CaMKII-hA_2A_R) animals confirms an age-associated decrease in the threshold for LTD induction.

The fact that in aged CA1 pyramidal neurons there is an increased duration of NMDAR-mediated responses [106] which display an altered Ca^2+^ metabolism typified by larger increases upon repeated stimulation [81, 107] further strengthens our hypothesis. This increase in Ca^2+^ observed in aging can lead to CREB dephosphorylation due to an increase in calcineurin (PP2B) activity, strongly suggesting differential phosphatases and kinases activation as a key mediator in these impairments [108, 109]. Alterations in phosphatases and kinases could directly account for the observed LTD-to-LTP shift. Importantly, we not only showed that susceptibility to induction of LTD is associated with memory impairments in aging, but also that the LTD magnitude could be positively correlated with behavior performance, consistent with previous data [57, 110].

The fact that an acute A_2A_R blockade is sufficient to rescue the LTD-to-LTP shift favors the hypothesis that A_2A_R blockade reestablishes the physiological signaling of adenosine, rather than the receptor expression, which is unlikely to occur at such a short time frame. Accordingly, we have prior data showing that chronic KW6002 treatment rescues cognitive and synaptic impairments induced by stress, without altering A_2A_R levels [45].

There is a growing awareness of AD beginning as a synaptic pathology [111], but very little is known concerning LTD in these animals [112–114]. We now demonstrate that, as our Tg(CaMKII-hA_2A_R) model, APP/PS1 animals exhibit this LTD-to-LTP shift. Alterations in NMDAR have been consistently linked to AD pathology [59, 115–119] that we now report to be dependent on A_2A_R activation. This abnormal A_2A_R/NMDAR crosstalk may underlie the efficiency of A_2A_R blockade in reverting memory deficits in animal models of AD [26, 35, 39].

The combined evidence of an increased A_2A_R expression in hippocampal neurons from humans (aged individuals and AD patients) and from animal models of these physiopathological conditions (Fig. 1; [26, 46, 68]) and the complete rescue of the LTD-to-LTP shift upon A_2A_R acute blockade stresses out A_2A_R as a putative pathological mediator involved in calcium dysfunction underlying age- and AD-related cognitive deficits, involving an aberrant recruitment of mGluR5/NMDAR coupled to an altered Ca^2+^ influx (see Supplementary Fig. 5 for a summary).

## Methods

### Human samples

The use of human samples was conducted in accordance with the Helsinki Declaration as well as national ethical guidelines. Protocols were approved by the Local Ethics Committee and the National Data Protection Committee. Human AD samples were provided by Valerie Buée-Scherrer (INSERM UMR-S1172 “Alzheimer & Tauopathies”, Lille Neurobank, Jean-Pierre Aubert Research Center Univ. Lille-Nord de France, France) or by Pedro Pereira and José Pimentel (Laboratório de Neuropatologia, Hospital de Santa Maria, CHLN, EPE, Lisboa, Portugal). Samples were collected from brains at 36 h post mortem. Aged and young human samples were collected by Beatriz S. da Silva (National Institute of Legal Medicine and Forensic Sciences, Coimbra, Portugal) and prepared by Paula M. Canas (CNC-Center for Neurosciences and Cell Biology, Univ. Coimbra, Coimbra, Portugal). After validation of their quality (Pliássova et al. 2016 [141]) young (20–40 years old), aged (60–75 years old) and AD (60–75 years old, Braak stages 5–6) human forebrain and hippocampus were used for histological analysis, Western blotting and qPCR as indicated.

### Animals

Animal procedures were performed in accordance with the European Community guidelines (Directive 2010/63/EU), Portuguese law on animal care (DL 113/2013), and approved by the *Instituto de Medicina Molecular* Internal Committee and the Portuguese Animal Ethics Committee (*Direcção Geral de Veterinária*). Environmental conditions were kept constant: food and water ad libitum, 21 ± 0.5 °C, 60 ± 10% relative humidity, 12 h light/dark cycles, 2 to 3 rats per cage or 3 to 4 mice per cage. Only male animals were used in all experiments. Mice were sacrificed by cervical dislocation and rats were sacrificed by decapitation after anesthesia under halothane atmosphere. Male Tg(CaMKII-hA_2A_R) Sprague-Dawley rats and their WT littermates with matched age (8–14 weeks old) or aged WT males (18–20 months old) were used for behavior and electrophysiology experiments. Male WT and APP/PS1 mice (11–12 months old) were used for electrophysiology experiments.

### Generation and maintenance of transgenic animals

Transgenic rats with an overexpression of human A_2A_R cDNA under the control of the Ca^2+^/calmodulin-dependent protein kinase II (CaMKII) promoter, Tg(CaMKII-hA_2A_R), were generated as previously described [30]. Expression of A_2A_R was achieved in forebrain areas, mainly in the hippocampus and cortex. Relevantly, the endogenous rA_2A_R mRNA levels were not modified in the hippocampus [30]. Furthermore, there was no changes in adenosine A_1_ receptor levels in the hippocampus of Tg(CaMKII-hA_2A_R) animals [30].

#### Genotyping

Transgenic rats were identified by PCR (30 cycles, 58  °C annealing temperature) of their genomic DNA isolated from ear biopsies by the use of the CaMKII-hA2AR transgene-specific primers and rat β-actin primers as an internal control (Invitrogen, USA; see Supplementary Table 1). APP/PS1dE9 transgenic mice on C57Bl6/J background have been described elsewhere [120]. Genotyping was done by PCR analysis of tail DNA (30 cycles, 60  °C annealing temperature) using transgene-specific primers (APP and PrP) and tau as an internal control (Supplementary Table 1).

### Oral administration of the drug

KW6002 (istradefylline), a selective A_2A_R antagonist [121, 122], was diluted in the drinking water (0.025% methylcellulose) and was orally administered to WT, Tg(CaMKII-hA_2A_R) and aged animals, being continuously available. The experimenter was blinded to genotype for the duration of KW6002 administration. The weight of the animals and the volume intake were assessed twice a week and the concentration of the solution was adjusted so that the drug intake was maintained at 5 mg kg^–1^ per day. The treatment started at 5–7 weeks of age in WT and Tg(CaMKII-hA_2A_R) and at 16 months of age in aged animals, and lasted for 1 month or 3 weeks, respectively, until sacrifice.

### RNA extraction and quantitative real-time PCR analysis (RT-qPCR)

Total RNA was extracted and purified using the RNeasy Lipid Tissue Mini Kit (Qiagen, Germany). RNA quality was assessed by NanoDrop 2000 (Thermo Scientific, USA) analysis (*A*_260_/*A*_280_ ≈ 2; *A*_260_/*A*_235_ > 1.8). Total RNA (2 μg) was reverse-transcribed using random primers and SuperScript™ First-Strand Synthesis System for RT-PCR (Invitrogen). RT-qPCR analysis was performed on a Corbett Rotor-gene 6000 apparatus (Qiagen, Germany) using Power SYBR Green PCR Master Mix (Applied Biosystems, UK), 0.2 µM of each primer and 1/20 dilutions of total cDNA (final concentration 0.4 ng/µl). The thermal cycler conditions were 10 min at 95 °C, 40 cycles of a two-step PCR, 95 °C for 15 s followed by 60 °C for 25 s with a final thermal ramp from 72 to 95 °C. Primer efficiencies (*E* = 1 ± 0.02) were obtained from standard curves of serial dilutions (slope and *R*^2^ around −3.3 and 0.99, respectively). The sequences of the primers used (all from Invitrogen, HPLC purified) are listed in Supplementary Table 1. Reference genes were PPIA (cyclophilin A) and β-actin for human tissues and PPIA, β-actin, Rpl13A and Pgk1 for rat tissue. Amplifications were carried out in triplicate in two independent runs, and according to the MIQE guidelines [123]. The relative expression of target genes was determined by the comparative CT method [124].

### In situ hybridization

The in situ hybridization technique was adapted from previously described methods [125]. The sections mounted on RNAse free poly-l-lysine-coated slides were fixed in freshly prepared 4% paraformaldehyde solution for 30 min and rinsed in phosphate-buffered saline (PBS: 130 mM NaCl, 7 mM Na_2_HPO_4_, 3 mM NaH_2_PO_4_). All sections were dehydrated and dipped for 3 min in chloroform. After air drying, the sections were incubated overnight at 42 °C with 0.35 × 10^6^ cpm per section of ^35^S-labeled probes diluted in hybridization buffer, which consisted of 50% formamide, 4xSSC (1xSSC: 0.15 M NaCl, 0.015 M sodium citrate, pH 7.4), 1 × Denhardt’s solution (0.02% polyvinylpyrrolidone, 0.02% bovine serum albumin (BSA), 0.02% Ficoll, 1% sarcosyl, 0.02 M sodium phosphate at pH 7.4, 10% dextran sulfate, 500 μg/ml yeast tRNA, 100 μg/ml salmon sperm DNA, and 60 mM dithiothreitol). After hybridization, the sections were rinsed for 4 × 15 min in 1xSSC at 55 °C, dehydrated and covered with Hyperfilm-βmax film (Amersham, Belgium) for 2 or 3 weeks. The oligonucleotide probes were synthesized using an Applied Biosystems 381A DNA synthesizer or Eurogentec (Belgium) with a GC to AT ratio between 45 and 65%. The human A_2A_R oligonucleotide probe (CAGCCCTGGGAGTGGTTCTTGCCCTCCTTTGGCTGACC-GCA) is complementary to nucleotides 123–166 in a partial human cDNA sequence [126] and has been previously used on human brain sections [127]. The rat A_2A_R probe (CCGCTCCCCTGGCAGGGGCTGGCTCTCCATC-TGCTTCAGCTG) is complementary to nucleotides 604–645 of the rat cDNA sequence [128]. Oligonucleotides were labeled with α-^35^S dATP (DuPont-NEN, Belgium) at their 3' end by terminal DNA deoxynucleotidylexotransferase (Gibco, Belgium) and purified with a G50 column (Pharmacia, Belgium) according to the manufacturer’s instructions.

### Behavioral assessments

Rats were first handled for 5 days prior to behavioral tests. Mazes were cleaned with a 30% ethanol solution between each animal. Animals were randomized prior to behavioral assessment and the experimenter blinded to genotype for the duration of behavioral testing. All behavioral tests were performed during the light phase between 8:00 a.m. and 6:00 p.m. in a sound attenuated room.

#### Morris water maze (MWM)

Spatial memory ability was evaluated in the MWM test, as previously described [45]. WT and Tg(CaMKII-hA_2A_R) animals were randomized and the experimenter blinded to genotype for the duration of behavioral testing. The test was performed over the course of 5 consecutive days and consisted of a 4-day acquisition phase and a 1 day probe test. The test was performed in a circular pool (1.8 m diameter, 0.6 m height), filled with water opacified with non-toxic black paint and kept at 25 ± 2 °C. A round 8-cm in diameter platform was hidden 1 cm beneath the surface of the water at a fixed position. Four positions around the edge of the tank were used, dividing the tank into four quadrants: target quadrant (T, quadrant where the platform was hidden), left quadrant (L, quadrant on the left of the target quadrant), right quadrant (R, quadrant of the right of the target quadrant) and opposite quadrant (O, quadrant on the opposite side of the target quadrant). During the acquisition phase, each animal was given four swimming trials per day (30-mins inter-trial interval). A trial consisted of placing the animal into the water facing the outer edge of the pool and allowing the animal to explore and reach for the hidden platform. If the animal reached the platform before 60 s, it was allowed to remain there for 10 s. If the animal failed to find the target before 60 s, it was manually guided to the platform, where it was allowed to remain for 20 s. After the end of each trial, animals were removed from the pool and placed back to their home cages beneath heat lamps in order to prevent temperature loss. During the probe test, the platform was removed and animals were allowed to swim freely for 60 s while recording the percentage of time spent on each quadrant. The latency to find the platform during the acquisition phase and the percentage of time in the platform quadrant during the probe test were recorded and analyzed using the Smart 2.5 tracking system (PanLab, Barcelona) to evaluate hippocampal-dependent memory. Swimming speed was also registered, as a measure of possible motor deficits that could interfere with the ability to perform the task.

#### Y-maze behavior test

Short-term reference memory was assessed in a spontaneous novelty-based spatial preference Y-maze test. The Y-maze was performed in a two-trial recognition test in a Y-shaped maze with three arms (each with 35 cm length x 10 cm width x 20 cm height), angled at 120° and with opaque walls. Different cues were placed on the surrounding walls. Allocation of arms was counterbalanced within each group. On the first trial (learning trial), the animal explored the maze for 10 min with only two arms opened (“start” and “other” arm). Access to the third arm of the maze (“novel” arm) was blocked by an opaque door. The rat was then removed from the maze and returned to its home cage. After 1 h, the animal was placed again in the “start” arm of the maze, the door of the “novel” arm was removed and the rat was allowed to explore the maze for 5 min (test trial). Rat tracings were continuously monitored by an automated tracking system (Smart 2.5, PanLab, Barcelona). Preference for the novel arm is considered a measure of short-term reference memory. To exclude the possible confounding effect of alterations of locomotor activity, we used the frequency of entrance into the arms (number of transitions) as an indirect indicator of the general locomotor activity.

### Electrophysiology experiments

After decapitation, the brain was rapidly removed and the hippocampi were dissected free in ice-cold Krebs solution, which is composed of (mM): NaCl 124; KCl 3; NaH_2_PO_4_ 1.25; NaHCO_3_ 26; MgSO_4_ 1; CaCl_2_ 2 and d-glucose 10, continuously gassed with 95% O_2_ and 5% CO_2_, pH 7.4. Transverse hippocampal slices (400 µm thick) were obtained with a McIlwain tissue chopper and field excitatory postsynaptic potentials (fEPSPs) were recorded in the *stratum radiatum* of the CA1 area at 32 °C, as previously described [45]. Tested drugs, SCH 58261 (50 and 100 nM), caffeine (30 µM), MPEP (5 µM) or AP5 (15, 50 and 100 µM), were added to the Krebs superfusion solution (3 ml/min) after obtaining a stable 10 min baseline. LTD was induced as previously [39] with three trains of 2 Hz during 10 min separated by a 10-min interval, or 1200 pulses at 0.5, 1, and 2 Hz, with baseline fEPSPs of 0.5 mV/ms. The magnitude of synaptic plasticity was calculated as percentage of change of fEPSP slope 50–60 min after LTD induction compared to baseline fEPSP (10 min before LTD induction). Recordings were performed at 32 °C, 3 ml/min.

For patch-clamp recordings, transverse hippocampal slices (300 µm) were cut in an oxygenated ice-cold solution containing (mM): 234 sucrose, 2.5 KCl, 1.25 NaH_2_PO_4_, 0.5 CaCl_2_, 10 MgSO_4_, 11 glucose, 26 NaHCO_3_. They were incubated at 37 °C for 1 h and then maintained at room temperature (RT) for 0.5–5 h in an oxygenated physiological solution (ACSF) containing (in mM): 119 NaCl, 2.5 KCl, 1.25 NaH_2_PO_4_, 2.5 CaCl_2_, 1.3 MgSO_4_, 11 glucose, 26 NaHCO_3_, pH 7.4. For recording, slices were transferred into a submerged recording chamber perfused with oxygenated ACSF at 3 ml/min at 32 °C and visualized under IR-DIC on a slidescope at x60 magnification (Scientifica Ltd., UK). Recordings were made using a patchstar micromanipulator (Scientifica Ltd.) connected to a Multiclamp700B amplifier and Digidata 1440 acquisition system controlled by the pClamp 10 software (Axon instruments, Molecular Devices Ltd., USA). Patch pipettes were made of borosilicate glass and shaped to a final resistance of approximately 5 MΩ.

#### Current clamp experiments

Whole-cell patch-clamp experiments were performed in the current clamp configuration [129] using a pipette solution containing (mM): 135 gluconic acid (potassium salt: K-gluconate), 5 NaCl, 2 MgCl_2_, 10 HEPES, 0.5 EGTA, 2 ATP-Tris, and 0.4 Tris-GTP. After a tight seal ( > 1 GΩ) on the cell body of the selected neuron was obtained, whole-cell patch-clamp configuration was established, and cells were left to stabilize for approximately 2  min before recordings began. The resting (*V*_m_) membrane potential was first measured in the absence of any spontaneous firing, and only cells with *V*_m_  −55  mV were considered. We then injected a minimum amount of current (150 pA) to stimulate a sustained firing that we recorded for a few minutes. Using this tonic firing, we measured the fast and medium afterhyperpolarization potentials (fAHP and mAHP, respectively) (Supplementary Fig. 3a, b). The maximum rising slope, the overshoot and the action potential (AP) half-width were also considered (Supplementary Fig. 3a, b). The half-width value was calculated considering the AP width measured at 50% of the peak amplitude. These AP parameters were estimated without taking into account the voltage drop across the pipette resistance. To study the relationship between firing frequency and current input (Supplementary Fig. 3c, d), we first adjusted the membrane potential to −60  mV and then injected 16 pulses of increasing intensity (from 100 to 850 pA, 200  ms duration). We also used these recordings to measure the instantaneous firing frequency at the beginning (onset frequency, *f*_o_, corresponding to the firing frequency measured between the first and second APs in the spike train) and at the end of the spike train (steady-state frequency, *f*_ss_, corresponding to the firing frequency measured between the last two APs in the spike train) (Supplementary Fig. 3c). By plotting *f*_o_ and *f*_ss_ as a function of injected current (Supplementary Fig. 3e, f), we obtained information on the spike frequency adaptation of these neurons. To quantify the inward rectification time-dependent potential, we first adjusted the membrane potential (*V*_h_) to −60  mV and injected 20 pulses of increasing intensity (from −100 pA to −2  nA, 600  ms duration). During the pulse, we observed that the hyperpolarization reached a maximum value (peak) and then decreased to stabilize to a steady-state value (Supplementary Fig. 3g). We plotted the difference between the peak and the steady-state values as a function of injected current to obtain indirect information on the hyperpolarization-activated inward current (Ih) (Supplementary Fig. 3h).

#### Voltage-clamp experiments

Whole-cell patch-clamp experiments were performed in the voltage-clamp configuration [117, 130] using a pipette solution containing (in mM): 117.5 cesium methanesulfonate, 15 CsCl, 10 tetraethylammonium chloride (TEACl), 8 NaCl, 10 HEPES, 0.25 EGTA, 4 MgATP, 0.3 NaGTP; the pH was adjusted to 7.3 with CsOH. For all experiments, slices were superfused with the oxygenated ACSF at 32  °C in the continuous presence of 50  µM picrotoxin (dissolved in Dimethylsulfoxide (DMSO), Sigma-Aldrich, France) to block GABAergic transmission. The Schaffer collateral pathway was stimulated at 0.10  Hz using electrodes (glass pipettes filled with ACSF) placed in the *stratum radiatum*. After a tight seal ( > 1 GΩ) on the cell body of the selected neuron was obtained, whole-cell patch-clamp configuration was established, and cells were left to stabilize for approximately 2  min before recordings began. To measure the paired-pulse ratio (PPR), two stimuli were delivered with inter-spike intervals between 50 and 200  ms. PPRs were calculated as the ratio between the peak amplitude of EPSC_2_ and of EPSC_1_ (20 sweeps average per inter-spike interval) (Supplementary Fig. 3i). To calculate the AMPAR/NMDAR ratio (Fig. 2f), cells were held at −65  mV to record AMPAR EPSCs and at  + 40  mV to record NMDAR EPSCs. AMPAR EPSCs amplitudes were calculated by averaging 30 consecutive EPSCs recorded at −65  mV and measuring the peak compared to the baseline. NMDAR EPSCs amplitudes were calculated by averaging 30 consecutive EPSCs recorded at  + 40  mV and measuring the amplitude 60 ms after EPSC onset compared to the baseline. Before starting *I*-*V* relationship measurements, stimulus intensity was set to evoke an EPSC of approximately 100 pA at −60  mV, normalizing the response and thus the number of recruited fibers. Liquid junction potential was not corrected for whole-cell voltage-clamp recordings. For EPSC_NMDAR_ *I*-*V* relationship measurements (Fig. 2g), pharmacologically isolated NMDAR EPSCs were obtained in the presence of 6,7-dinitroquinoxaline-2,3-dione (DNQX, 100  µM dissolved in 1% DMSO, Sigma-Aldrich). NMDAR EPSCs amplitudes were calculated by averaging 15 consecutive EPSCs recorded at voltages ranging from −70  mV to  + 40  mV in 10  mV steps. *I*–*V* relationships were normalized to the NMDAR EPSC amplitude at  + 40  mV (as + 1). AMPAR *I–V* relationships were recorded using an identical procedure, but in presence of R-2-amino-5-phosphonopentanoate (AP5, 50  µM dissolved in DMSO, Sigma-Aldrich) and were normalized to the AMPAR EPSC amplitude at −70  mV (as -1). The decay time of pharmacologically isolated NMDAR EPSC, recorded from cells voltage clamped at  + 40  mV, was fit with a double exponential function, using Clampfit software, to calculate both slow and fast decay time constants, *τ*_slow_ and *τ*_fast_, respectively (Fig. 2i). The weighted time constant (*τ*_weighted_) was calculated using the relative contribution from each of these components, applying the formula: *τ*_w _ =  [(*a*_f_. *τ*_f_)  +  (*a*_s_. *τ*_s_)]/(*a*_f _ +  *a*_s_), where *a*_f_ and *a*_s_ are the relative amplitudes of the two exponential components, and *τ*_f_ and *τ*_s_ are the corresponding time constants (Fig. 2i).

### Primary neuronal cultures

Hippocampal neurons were cultured from 18 day Sprague-Dawley rat embryos (Harlan, Barcelona, Spain) as previously described [131]. Briefly, embryos were collected in Hank’s Balanced Salt Solution (HBSS, Corning, USA) and rapidly decapitated. Meninges were removed, and whole cortices (hippocampi and attached cortex) were dissociated and incubated for 15 min in HBSS with 0.025% trypsin. Cells were washed once with HBSS with 30% fetal bovine serum (FBS), centrifuged three times, re-suspended in Neurobasal Medium (Gibco–Life Technologies, USA) supplemented with 2% B-27 supplement, 25 μM Glutamate, 0.5 mM glutamine, and 2 U/ml penicillin/streptomycin, gently dissociated and filtered through a 70 μm strainer (VWR, USA). Cells were plated on poly-d-lysine-coated plates and grown for 14 days at 37 °C in a 5% CO_2_-humidified atmosphere in the previously described supplemented Neurobasal medium, in the absence of any positive selection for neurons.

### Transfection of primary neuronal cultures

At DIV (day in vitro) 13, neurons were transfected as previously described [132]. A 33 ± 4% efficiency of transfection was obtained. At DIV 14, Ca^2+^ imaging experiments and immunocytochemistry to confirm transfection were performed.

### Construct generation

Venus-A_2A_R construct was generated with the In-fusion HD Cloning Kit (Clontech Takara, USA). Venus and A_2A_R fragments were produced by using, respectively, the pair of primers 5'-GTTTAAACTTAAGCTTATGGTGAGCAAGGGCGAG-3' and 5'-GCTGCCCATGGTGGCCTTGTACAGCTCGTCCATG-3', and the pair of primers 5'-GCCACCATGGGCAGCAGC-3' and 5'-AAACGGGCCCTCTAGATCAGCTGGGGGCGAACTC-3'. PCR fragments were cloned into the vector pcDNA3.1( + ) linearized with *Hin*dIII and *Xba*I, and the resulting construct was verified by DNA sequencing (GATC Biotech, Germany).

### Ca^2+^ imaging

Primary neuronal cultures were plated at a density of 50 × 10^3^ cells per well in 35 mm glass bottom culture dishes (MatTek Corporation, USA) previously coated with poly-d-lysine. At DIV 14, neurons were loaded with Fura-2 AM (5 µM, in external physiological solution with the following composition in mM: NaCl 125, KCl 3, NaH_2_PO_4_ 1.25, CaCl_2_ 2, MgSO_4_ 1, d-( + )-glucose 10 and HEPES 10; pH 7.4 adjusted with NaOH) and incubated at 37 °C for 1 h. Cells were then placed on a heated chamber installed in an inverted microscope with epifluorescent optics and equipped with a high speed multiple excitation fluorimetric system (Lambda DG4, with a 175 W Xenon arc lamp). Fura-2 AM loaded neurons were sequentially excited both at 340 and 380 nm, for 250 ms at each wavelength, and the emission fluorescence was recorded at 510 nm with a charge-coupled device (CDD) camera. Experiments were performed on cells with a baseline fluorescence ratio around 0.5, which corresponds approximately to a [Ca^2+^]i of about 100 nM, considered the normal [Ca^2+^]_I_ [133, 134]. Cells with a baseline fluorescence ratio above 1 were discarded. Experiments were performed at 37 °C in a 5% CO_2_-humidified atmosphere. Drugs were applied directly to the cells medium. All cells were challenged with ionomycin (a Ca^2+^ ionophore; 2 µM) at the end of the experiment and only those that responded were included, confirming neuronal viability. Image data were recorded and analyzed using the MetaFluor software (Universal Imaging, West Chester, PA, USA).

### Immunocytochemistry

Twenty-four hours after transfection, primary neurons were washed with PBS and fixed with 4% paraformaldehyde for 10 min at RT, followed by a permeabilization step with 0.5% Triton X-100 (Sigma–Aldrich) for 20 min at RT. After blocking in 10% FBS for 30 min, the cells were incubated with mouse anti-A_2A_R primary antibody (1:100, mouse monoclonal, mab70192, Covalab, France) overnight at 4 °C. After a 30-min washing with PBS, cells were incubated with the secondary antibody Alexa Fluor 568 goat anti-mouse IgG (Life Technologies-Invitrogen) for 1 h at RT. Finally, the cells were stained with Hoechst 33258 (1 mg/mL, Life Technologies;1:5000 in PBS) for 5 min and mounted in Dako mounting medium. Z-stack images at 63 × magnification were acquired with a Zeiss LSM 880 confocal microscope.

### Immunohistochemistry

Brains were removed, stored in formaldehyde 4% aqueous solution (VWR, USA) for 3 days, embedded in paraffin, and cut into coronal sections of 2 μm. Slides were deparaffinized, rehydrated and antigen retrieval was performed by microwave heating in 0.01 M citrate buffer pH = 6.0. For fluorescence analysis, slices were then incubated with primary antibodies selective for A_2A_R (1:100, mouse monoclonal, mab70192, Covalab) and GFAP (1:250, rabbit polyclonal, G9269, Sigma-Aldrich), MAP2 (1:500, rabbit polyclonal, ab32454, Abcam, UK), SNAP25 (1:5000, rabbit polyclonal, S9684, Sigma-Aldrich), synaptophysin (1:200, mouse monoclonal, S7568, Sigma-Aldrich) or PSD95 (1:100, rabbit polyclonal, D27E11, Cell Signaling Technology, UK) overnight at RT and washed for 20 min with PBS before being incubated overnight at RT with secondary antibodies (Alexa Fluor 488 donkey anti-rabbit and Alexa Fluor 568 donkey anti-mouse 1:400, Life Technologies, USA). After washing for 20 min, the sections were incubated with Hoechst 33342 (12 μg/ml final concentration; Thermo Scientific, USA), washed once and mounted in Dako Mounting Medium (Agilent, USA). Z-stack images at 63 × magnification were acquired with a Zeiss LSM 880 Confocal Microscope with Airyscan. The images were acquired with a 63 × objective, model Plan-Apochromat, a numerical aperture of 1.40 and a working distance of 0.19 mm. The images were acquired with a voxel size of *x*:132 nm, *y*:132 nm, *z*:316 nm, and the point spread function (PSF) monitored with beads of 175 nm was *XY* = min 205 ± 4 nm, max 234 ± 3 nm, and *Z* 478 ± 30 nm (emission wavelength 525 nm). Co-localization analysis between A_2A_R and SNAP25/PSD95 was performed in single plans with co-localization threshold tool in Fiji software [135], which calculates several co-localization parameters and generates an image with co-localized pixels stained in white (Supplementary Fig. 2a, b). Compositional images of hippocampal formation were produced by tile stitching of images at 10 × magnification acquired using Zeiss Axio Observer Widefield microscope. For human samples, coronal sections were stained with an anti-A_2A_R (1:100, mab70192, Covalab) and developed using amplification (NovoLink™ Polymer Detection System, Leica Biosystems, Germany) and horseradish peroxidase–diaminobenzidine (HRP-DAB) detection systems. In parallel, an age-matched control section was used as a negative control, where no primary antibody was used. Samples were then mounted in Entellan® mounting medium (Sigma-Aldrich). Optical density was measured using ImageJ software in one field of 20 × magnification and three fields of 40 × magnification.

### Electron microscopy

Tg(CaMKII-hA_2A_R) animals were anesthetized using isoflurane and fixed using perfusion pump with 0.1 M phosphate buffer containing 2% paraformaldehyde and 0.2% glutaraldehyde. After removal of the brain, 500 µm slices of hippocampus were collected using a Vibratome (Leica, Germany). Immunoelectron microscopy of hippocampal slices was performed according to Tokuyasu [136]. Slides were chemically fixed in 0.1 M phosphate buffer containing 2% paraformaldehyde and 0.2% glutaraldehyde, embedded in gelatine (Royal® food grade gelatine) and cryo-preserved in 2.3 M sucrose. Gelatine blocks were frozen in liquid nitrogen and sectioned at −120 °C using an cryo-ultramicrotome (UC7 and FC7, Leica) to generate 70 nm sections, sections were collected and thaw in a mixture of 2.3 M sucrose and 2% methylcellulose. Immuno-labeling was done in 1% bovine serum albumin and 0.8% gelatine from cold water fish skin in PBS with polyclonal rabbit anti-A_2A_R primary antibody (pab70273, 1:50, Covalab) and 15 nm gold coupled Protein A (CMC Utrecht, 1:50). After immuno-labeling, the sections were stained and mounted in a mixture of 3% (aq.) uranyl acetate and 2% methylcellulose. Images were taken using a Hitachi H-7650 electron microscope at 100 kV acceleration. We counted the immunogold particles in 40 micrographs (total of 72 synapses) of the CA1 area of Tg(CaMKII-hA_2A_R) animals (according to ref. [137]) and evaluated blindly by two pathologists. We found an average of 2.4 particles/synapse. The gold labeling in synapses elements was categorized into pre or post ( < 30 nm within the active zone) and perisynaptic ( < 30 nm outside the active zone). No particles were found in the nucleus.

### Fractionation

Subcellular fractionation was performed as described previously [138]. Briefly, WT and Tg(CaMKII-hA_2A_R) frozen hippocampi were homogenized with Potter in a buffer containing sucrose 0.32 M and HEPES 10 mM. After centrifugation (1000 g for 10 min), the pellet was dissolved in a buffer containing HEPES 4 mM and EDTA 1 mM. After centrifugation (12,000 × *g* for 20 min), the pellet was dissolved in a buffer containing HEPES 20 mM, NaCl 100 mM, triton X-100 0.5%. After centrifugation (12,000 × *g* for 20 min), the supernatant is the non-postsynaptic density membrane fraction (non-PSD95-enriched fraction), as confirmed by the detection of enriched SNAP25 and the absence of PSD95 (Fig. 1h). The pellet was dissolved in a buffer containing HEPES 20 mM, NaCl 0.15 mM, triton X-100 1%, deoxycholic acid 1%, SDS 1% and centrifuged for 15 min at 10,000 × *g*. The supernatant is the postsynaptic density membrane fraction (PSD95-enriched fractions), as demonstrated by the detection of enriched PSD95 and sparse SNAP25. Equal volumes of non-PSD95 and PSD95-enriched fractions were diluted in sample buffer (see western blotting section) and denatured by heating to 65 °C for 20 min and used for western blot analysis.

### Western blotting

Tissue was homogenized by sonication using RIPA buffer (50 mM Tris, 1 mM EDTA, 150 mM NaCl, 0.1% SDS, 1% Tergitol-type NP-40, pH 8.0). The protein concentration was determined using a BioRad DC Protein assay kit [based on ref. [139])]. The appropriate volume of each sample was diluted in water and sample buffer (70 mM Tris pH 6.8, 6% glycerol, 2% SDS, 120 mM dithiothreitol and 0.0024% Bromophenol blue). The samples were denatured at 65 °C for 20 min. Based on the protocol of Towbin et al. [140], samples and molecular weight markers were separated by sodium dodecyl sulfate polyacrylamide gel electrophoresis (10% for resolving and a 5% for stacking gels) in denaturing conditions and electro-transferred to Polyvinylidene fluoride (PVDF) membranes (GE Healthcare, UK). Membranes were blocked with 3% BSA in TBS-T 0.1% (Tris-buffered saline with 0.1% Tween-20 solution, 200 nM Tris, 1.5 M NaCl) for 1 h and incubated with primary antibody (diluted in TBS-T, 3% BSA and 0.1% NaN_3_) overnight at 4 °C. Primary antibodies were mouse anti-A_2A_R (1:2000, 05-717, Upstate/Millipore, Germany), rabbit anti-SNAP25 (1:10,000, S9684, Sigma), rabbit anti-pan-cadherin (1:20,000, ab6529, Abcam), rabbit anti-PSD95 (1:1000, D27E11 Cell Signaling Technology) and mouse anti-α-tubulin (1:1000, sc-8035, Santa Cruz Biotechnology, USA). After three washing periods of 10 min with TBS-T, membranes were incubated with horseradish peroxidase (HRP)—conjugated anti-mouse or anti-rabbit secondary antibodies (1:10 000; Santa Cruz Biotechnology) (in 5% nonfat dry milk) for 1 h at RT. After 30 min of washing with TBS-T, chemiluminescent detection was performed with Enhanced chemiluminescence (ECL) western blotting detection reagent (GE Healthcare) using X-Ray films (Fujifilm, Japan). Optical density was determined with Image-J software and normalized to the respective pan-cadherin or tubulin band density.

### Drugs

The A_2A_R selective antagonist, 2-(2-furanyl)-7-(2-phenylethyl)-7H-pyrazolo[4,3-e][1,2,4]triazolo[1,5-c]¬pyrimi¬din-5-amine (SCH 58261) and the A_2A_R selective agonist 2-[*p*-(2-Carboxyethyl)-phenylethylamino]-5’-*N*-ethylcarboxamidoadenosine (CGS 21680) were purchased from Tocris (UK). GABA receptor antagonist picrotoxin, AMPA receptor antagonist 6,7-dinitroquinoxaline-2,3-dione (DNQX) and NMDA receptor antagonist (2 *R*)-amino-5-phosphonovaleric acid (AP5) were purchased from Sigma-Aldrich. mGluR5 antagonist 6-Methyl-2-(phenylethynyl)-pyridine hydrochloride (MPEP) was purchased from Enzo Life Sciences (USA). These drugs were diluted in the assay solution from 5 mM or 1 mM stock aliquots made in DMSO or water stored at –20 °C. All other reagents used were of the highest purity available either from Merck or Sigma–Aldrich.

### Statistical analysis

All statistical analyses were performed with GraphPad Prism software. Values are presented as mean ± s.e.m. in figure legends. Statistical analyses were designed using the assumption of normal distribution and similar variance among groups, as previously tested. Statistical comparisons included two-sided unpaired *t*-test, one or two-way ANOVA followed by a Bonferroni’s or Tukey’s multiple comparison post hoc tests as specified in the figure legends. *P*-values of < 0.05 were considered to be statistically significant. The sample size was determined based on Power Analysis or similar experiments carried out in the past. Power Analysis was performed using G-power in order to estimate the number of animals required, for a signal-to-noise ratio of 1.4 and 80% to 90% power assuming a 5% significance level.

### Data availability

For detailed information on experimental design please see the provided Reproducibility Checklist. Full-length gels and blots with molecular weight standards are provided in Supplementary Fig. 6. All the software used to data analysis is commercially available and the respective information is provided in each respective section. The data that support the findings of this study are available from the corresponding authors upon reasonable request.

## Electronic supplementary material

Supplementary Information

A2AR activation increases intracellular Ca2+ levels in primary neuronal cultures transfected with Venus-A2AR construct
