# Supplementary material for: Age-related shift in LTD is dependent on neuronal adenosine A2A receptors interplay with mGluR5 and NMDA receptors
Source: Mol Psychiatry. 2018 Jun 27;25(8):1876–900. doi: 10.1038/s41380-018-0110-9 (PMC7387321; doi:10.1038/s41380-018-0110-9)
Supplement: Supplementary file 1 — Supplementary Information [file 41380_2018_110_MOESM1_ESM.pdf]

Supplementary Information

Supplementary Figure 1

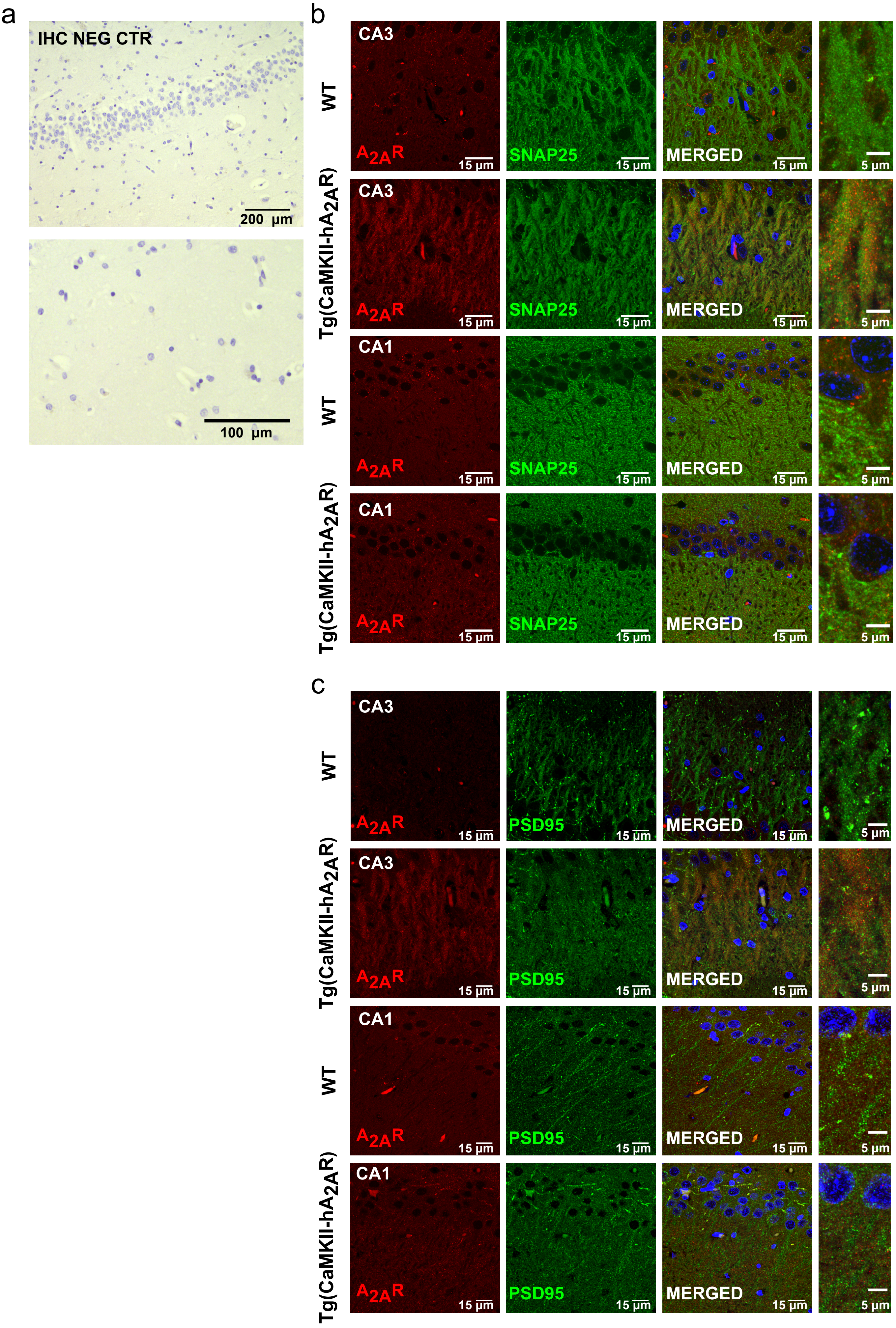

Supplementary Figure 1. Immunohistochemical analysis of $A_{2A}R$, SNAP25 and PSD95 in WT and Tg(CaMKII-h$A_{2A}R$) hippocampal slices. (**a**) Immunohistochemistry negative control of aged slices does not show non-specific $A_{2A}R$ staining. (**b**) Z-stack maximum intensity projection images taken at 63x magnification in CA1 and CA3 areas of hippocampus are presented (scale bars: 15 μm and 5 μm). Nuclei are stained with Hoechst (blue), $A_{2A}R$ are labeled with red and SNAP25 positive cells are stained in green. (**c**) Z-stack maximum intensity projection images taken at 63x magnification in CA1 and CA3 areas of hippocampus are presented (scale bars: 15 μm and 5 μm). Nuclei are stained with Hoechst (blue), $A_{2A}R$ are labeled with red and PSD95 positive cells are stained in green.

Supplementary Figure 2

a

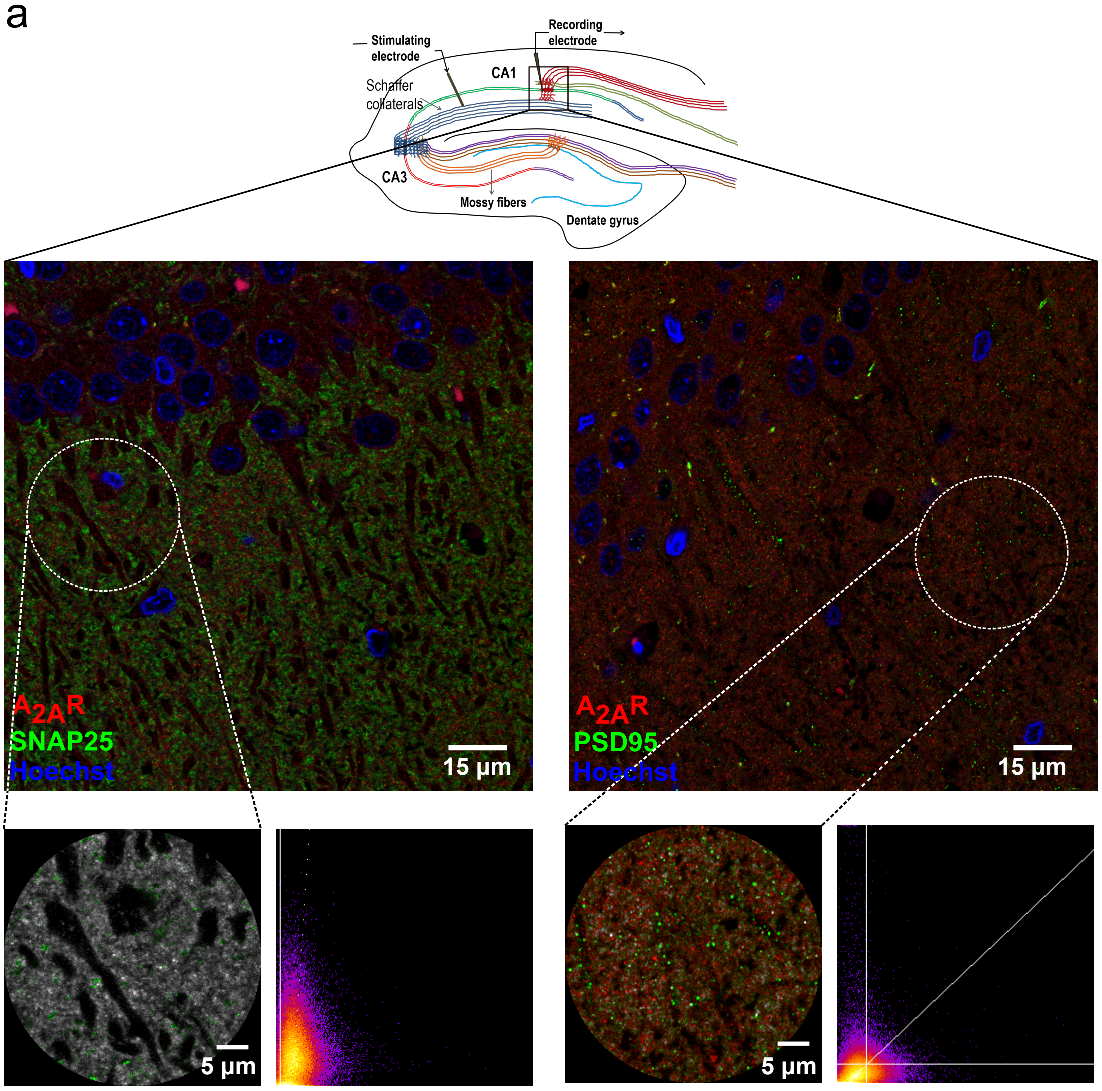

	tM $A_{2A}R$	tM SNAP25/ PSD95	Ncoloc	%$A_{2A}R$ Vol	%SNAP25/ PSD95 Vol	%$A_{2A}R$ Int	%SNAP25/ PSD95 Int	%$A_{2A}R$ int > thres	%SNAP25/ PSD95 int > thres
$A_{2A}R$ + SNAP25	1 ± 0	0,98 ± 0,01	66135 ± 428	98,29% ± 0,13	93,56% ± 0,61	99,42% ± 0,15	96,63% ± 0,46	99,71% ± 0,06	96,63% ± 0,46
$A_{2A}R$ + PSD95	0,22 ± 0,04	0,36 ± 0,03	4165 ± 437	20,32% ± 3,59	32,04% ± 2,51	10,38% ± 1,32	15,85% ± 0,84	21,28% ± 3,73	33,37% ± 2,09
p	< 0,0001	< 0,0001	< 0,0001	< 0,0001	< 0,0001	< 0,0001	< 0,0001	< 0,0001	< 0,0001

b

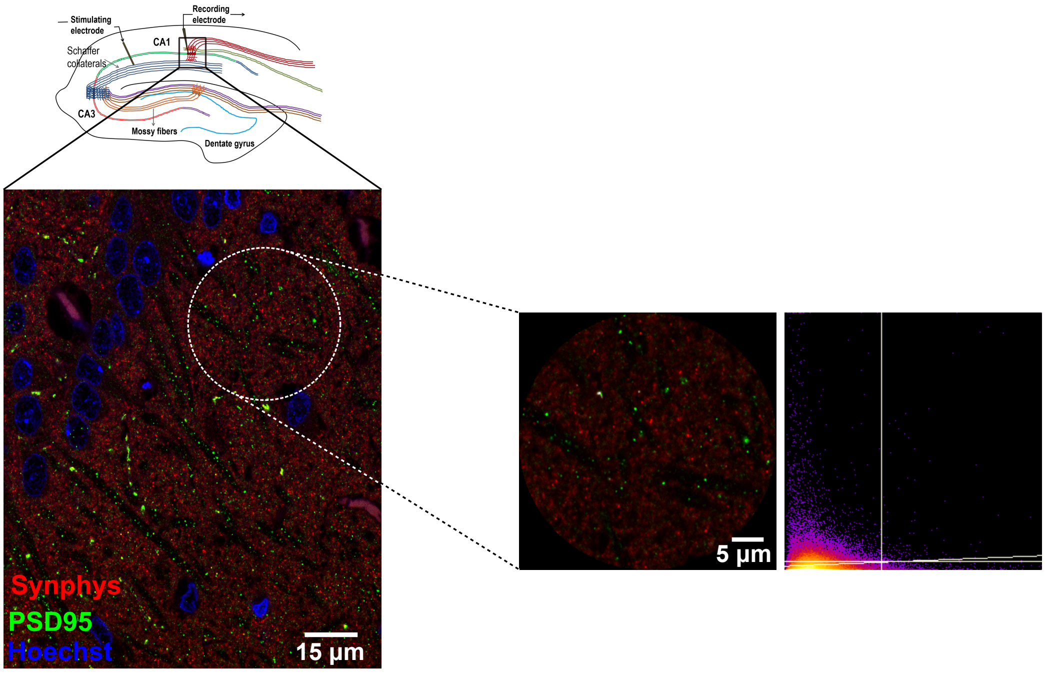

	tM synphys	tM PSD95	Ncoloc	%synphys Vol	%PSD95 Vol	%synphys Int	%PSD95 Int	%synphys int > thres	%PSD95 int > thres
Synphys + PSD95	0,25 ± 0,02	0,16 ± 0,06	1942 ± 894	23,84% ± 2,13	12,34% ± 5,12	6,27% ± 2,60	11,97% ± 4,78	24,19% ± 2,24	16,33% ± 6,19

Supplementary Figure 2. In Tg(CaMKII-hA_{2A}R) animals, A_{2A}R is mainly expresses pre-synaptically. (**a**) On top, images taken at 63x magnification in CA1 area of hippocampus are presented (scale bar: 15 µm). Nuclei are stained with Hoechst (blue), A_{2A}R are labeled with red and SNAP25 positive cells (left) or PSD95 positive cells (right) are stained in green. At center left, amplification of the upper image, where colocalized pixels are stained in white (scale bar: 5 µm). At center right, scatter plot (channel 1 (red; A_{2A}R) along the x-axis; channel 2 (green; SNAP25/PSD95) along the y-axis) with both the linear regression as well as the thresholds marked. We observe a stronger white labelling for A_{2A}R/SNAP25 than for A_{2A}R/PSD95, suggesting that A_{2A}R is mainly expressed pre-synaptically. On bottom, table with colocalization parameters measured in A_{2A}R/SNAP25 and in A_{2A}R/PSD95. *tM1* - channel 1: red, A_{2A}R; *tM2* - channel 2; green, SNAP25/PSD95 (Mander's adjusted to thresholds for each channel); *Ncoloc* (Number of colocalized voxels, number of voxels which have both channel 1 and channel 2 intensities above threshold); *%Ch1 Vol, %Ch2 Vol* (Percentage of voxels colocalized, number of voxels for each channel which have both channel 1 and channel 2 intensities above threshold, expressed as percentage of the total number of voxels for each channel above their respective thresholds); *%Ch1 Int, %Ch2 Int* (Percentage of intensity colocalized for each channel, this value is equal to the sum of the pixel intensities, with intensities above both channel 1 and channel 2 thresholds expressed as a percentage of the sum of all channel 1 intensities); *%Ch1 Int > thres, %Ch2 Int > thres* (percentage of intensities above threshold colocalized, for each channel, this value is equal to the sum of the pixel intensities with intensities above both channel 1 and channel 2 thresholds expressed as a percentage of the sum of all channel 1 intensities above the threshold for channel 1). We observe that for SNAP25-A_{2A}R, Mander's adjusted to threshold for both channels are close to 1, indicating colocalization, while this value is significantly lower for PSD95-A_{2A}R (A_{2A}R: 0.22 ± 0.04, PSD95: 0.36 ± 0.03). All the other colocalization parameters further confirm colocalization between A_{2A}R and SNAP25 and lack of colocalization between A_{2A}R and PSD95. (**b**) On top, image taken at 63x magnification in CA1 area of hippocampus is presented (scale bar: 15 µm). Nuclei are stained with Hoechst (blue), synaptophysin (synphys) is labeled with red and PSD95 positive cells are stained in green. At center left, amplification of the upper image, where colocalized pixels are stained in white (scale bar: 5 µm). At center right, scatter plot with both the linear regression as well as the thresholds marked. On bottom, table with colocalization parameters measured. We observe a lack of co-localization between synaptophysin and PSD95 signals.

Supplementary Figure 3

a

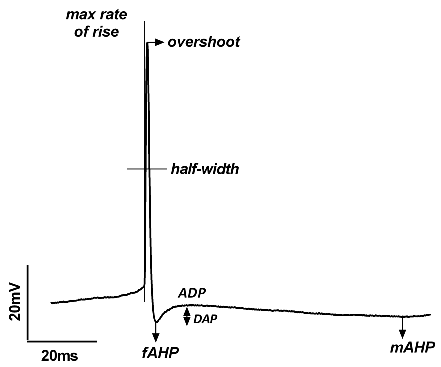

b

	Vm(mV)	fAHP(mV)	mAHP(mV)	Max. Rate of rise (mV/ms)	Overshoot (mV)	Half-width (ms)
WT (n=11)	-64,96±1,904	-52,99±1,245	-50,63±1,021	222,3±14,82	41,64±2,799	1,17±0,027
Tg(CaMKII-hA$_{2A}$R) (n=14)	-62,38±0,8481	-53,20±0,5734	-52,13±0,6308	251,7±16,49	41,85±2,893	1,10±0,027

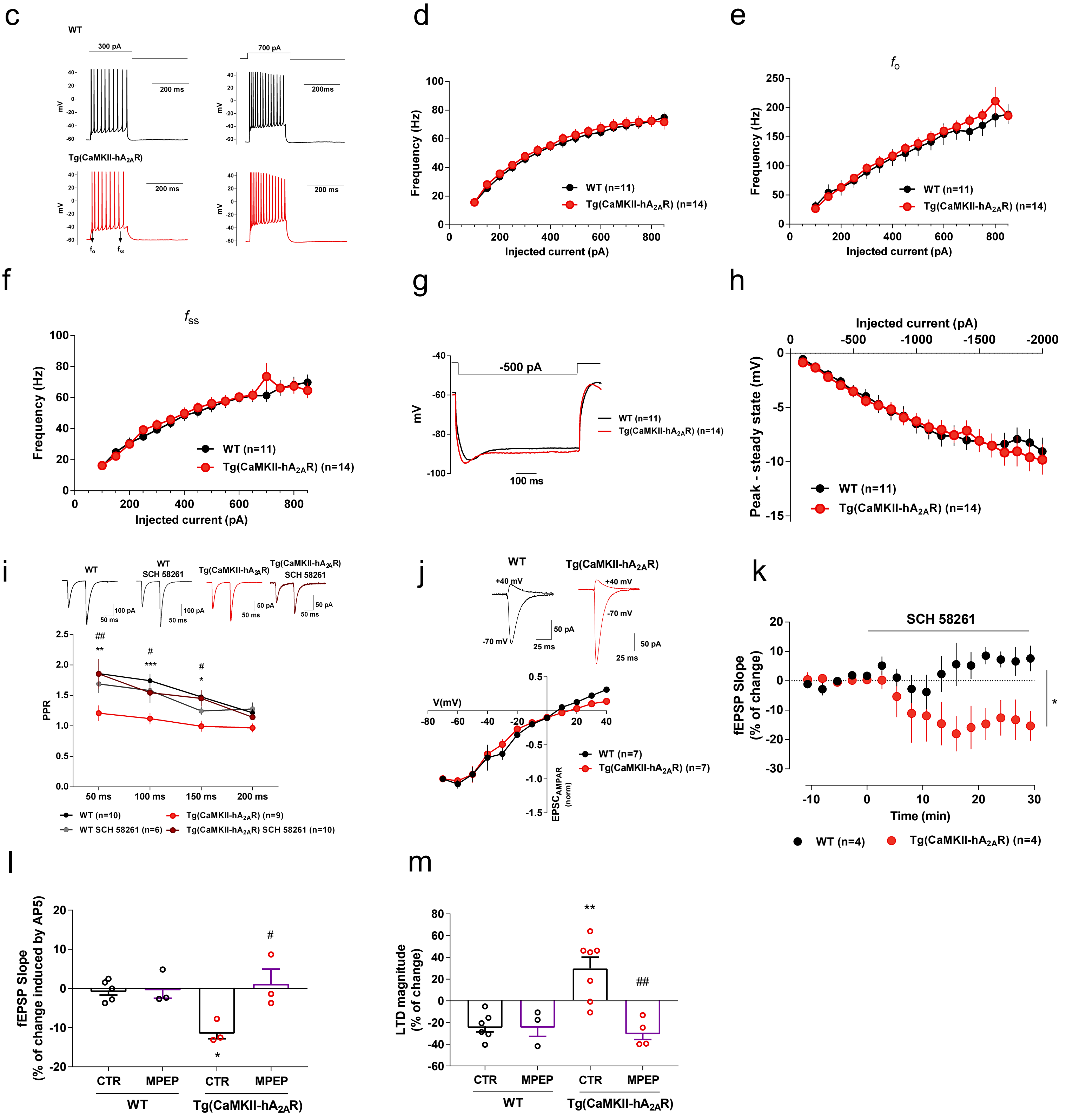

Supplementary Figure 3. Increased levels of $A_{2A}R$ enhance glutamate release probability. (**a**) Action Potentials parameters evaluated as summarized in (b). (**b**) Summary of action potential (AP) parameters measured during tonic firing in CA1 neurons from WT and Tg(CaMKII-hA_{2A}R) animals (n = 11 and 14, respectively). (**c**) Representative traces of AP trains in response to 300 and 700 pA current steps recorded from neurons from WT (top) and Tg(CaMKII-hA_{2A}R) (bottom) animals. (**d**) Mean firing frequency *versus* injected current for WT and Tg(CaMKII-hA_{2A}R) (n = 11 and 14, respectively). (**e**) The instantaneous firing frequency (f_o) measured between the first and second APs of the spike train plotted *versus* the injected current is similar in WT e Tg(CaMKII-hA_{2A}R) conditions (n = 11 and 14, respectively). (**f**) The instantaneous firing frequency (fss) measured between the last two APs of the spike train recorded in neurons from in WT and Tg(CaMKII-hA_{2A}R) is not different (n = n = 11 and 14, respectively). (**g**) Representative trace of the time-dependent inward rectification potential measured in WT and Tg(CaMKII-hA_{2A}R) during a 500 pA hyperpolarization current injection lasting 600 ms. (**h**) The difference between the peak and the steady-state values plotted *versus* the negative current amplitude (from -100 to -2000 pA) suggests that the hyperpolarization pattern is not changed in Tg(CaMKII-hA_{2A}R) (n = 11 and 14, respectively). (**i**) PPR values in neurons from WT and Tg(CaMKII-hA_{2A}R) animals, treated and non-treated with SCH 58261 (50 nM). For the interspike intervals between 50 and 150 ms, a decrease in PPR in Tg(CaMKII-hA_{2A}R) is observed, in comparison with the values obtained from WT neurons ($^*p < 0.05$, two-way ANOVA followed by a Bonferroni's multiple comparisons *post hoc* test) (n = 10 and 9, respectively). This decrease is lost when the interspike interval is 200 ms. When neurons from Tg(CaMKII-hA_{2A}R) animals are acutely treated with SCH 58261 (50 nM), the PPR values are completely reverted back to the WT levels ($^{\#}p < 0.05$ comparing to Tg(CaMKII-hA_{2A}R), two-way ANOVA followed by a Bonferroni's multiple comparisons *post hoc* test) (n = 10); representative traces of EPSCs with an inter-spike interval of 100 ms, for WT and Tg(CaMKII-hA_{2A}R) animals, with and without SCH 58261 perfusion. (**j**) Tg(CaMKII-hA_{2A}R) animals do not exhibit alterations in AMPAR activation (n = 7); representative traces of AMPAR EPSCs recorded at -70 mV and +40 mV. (**k**) SCH 58261 decreases basal transmission in Tg(CaMKII-hA_{2A}R) animals ($^*p < 0.05$, unpaired t test) (n = 4). (**l**) mGluR5 antagonist rescues NMDAR aberrant component on basal transmission ($^*p<0.05$ comparing to WT, $^{\#}p<0.05$ comparing to Tg(CaMKII-hA_{2A}R), two-way ANOVA followed by Bonferroni's multiple comparisons *post hoc* test) (n = 3). (**m**) MPEP rescues LTD-to-LTP shift in Tg(CaMKII-hA_{2A}R) animals ($^{**}p<0.01$ comparing to WT, $^{\#\#}p<0.01$ comparing to Tg(CaMKII-hA_{2A}R), two-way ANOVA) (n = 4), while no effect is observed in WT animals (n = 3). All values are mean ± SEM.

Supplementary Figure 4

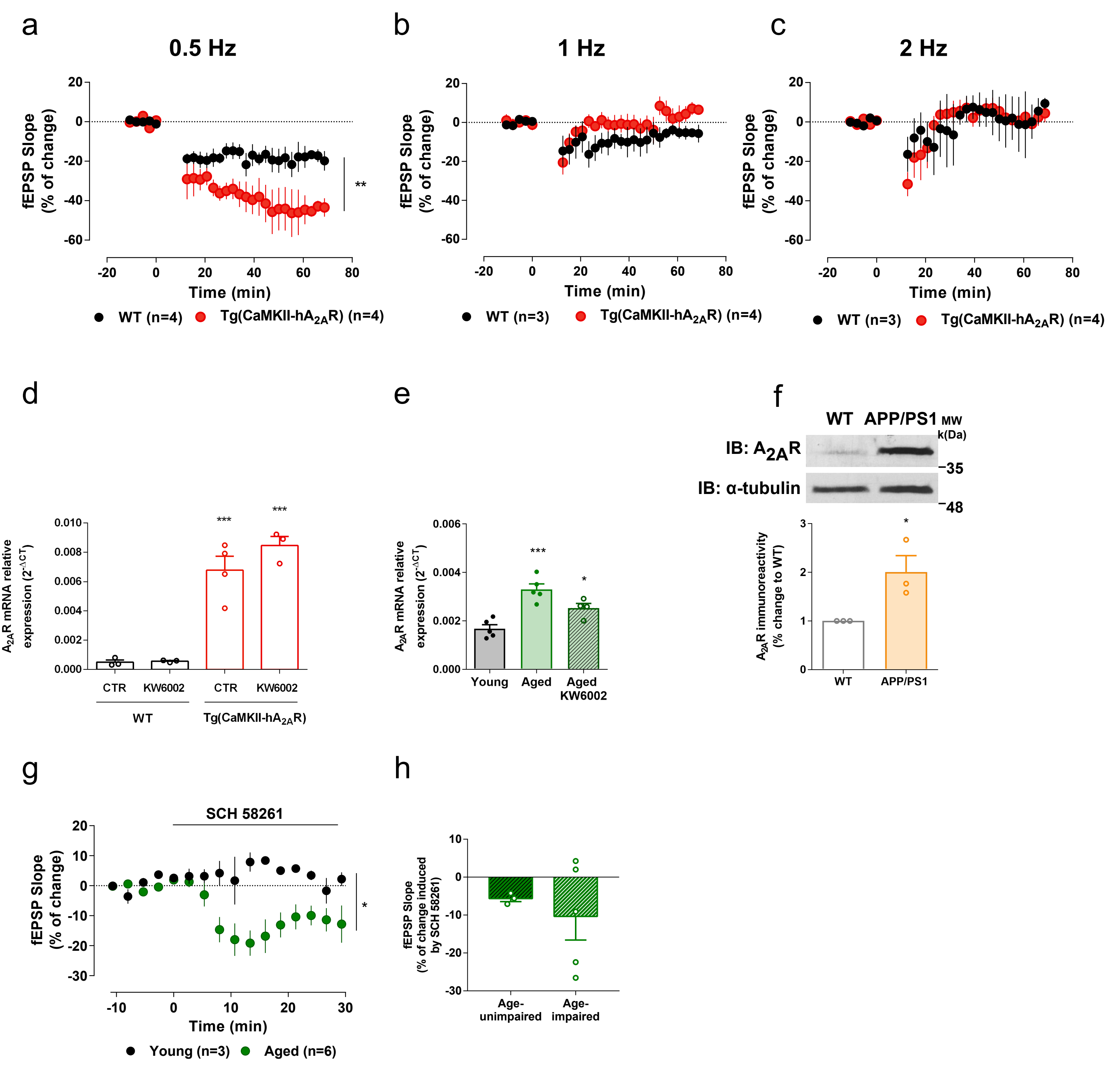

Supplementary Figure 4. Tg(CaMKII-hA_{2A}R) animals display alterations in LTD threshold. (**a**), (**b**), (**c**) Changes in fEPSP slope induced by LFS stimulation (0.5, 1 and 2 Hz, respectively) in WT and Tg(CaMKII-hA_{2A}R) animals (**$p < 0.01$, unpaired t test) (WT: n = 4, 3 and 3, respectively; Tg(CaMKII-hA_{2A}R): n = 4). (**d**) A_{2A}R mRNA relative expression in WT and Tg(CaMKII-hA_{2A}R) animals non-treated and treated with KW6002 (***$p < 0.001$ comparing to WT, two-way ANOVA followed by Bonferroni's multiple comparisons *post hoc* test) (WT: n = 3; Tg(CaMKII-hA_{2A}R): n = 4 and 3, respectively). (**e**) A_{2A}R mRNA relative expression in young and aged animals non-treated and treated with KW6002 (***$p < 0.001$, *$p<0.05$, comparing to young, one-way ANOVA) (n = 5, 5 and 4, respectively). (**f**) APP/PS1 mice displayed A_{2A}R hippocampal overexpression, as compared to WT animals (*$p < 0.5$, unpaired t test) (n = 3). (**g**) SCH 58261, 50 nM, decreases basal transmission in aged animals, while no effect is observed in young animals (*$p<0.05$, unpaired t test) (n = 6 and 3, respectively). (**h**) There is a tendency towards an increase in SCH58261 effect on basal transmission in age-impaired animals (n = 5), when compared to age-unimpaired animals (n=3). Uncropped gels and blots with molecular weight standards are provided in Supplementary Figure 6. All values are mean ± SEM.

Supplementary Figure 5

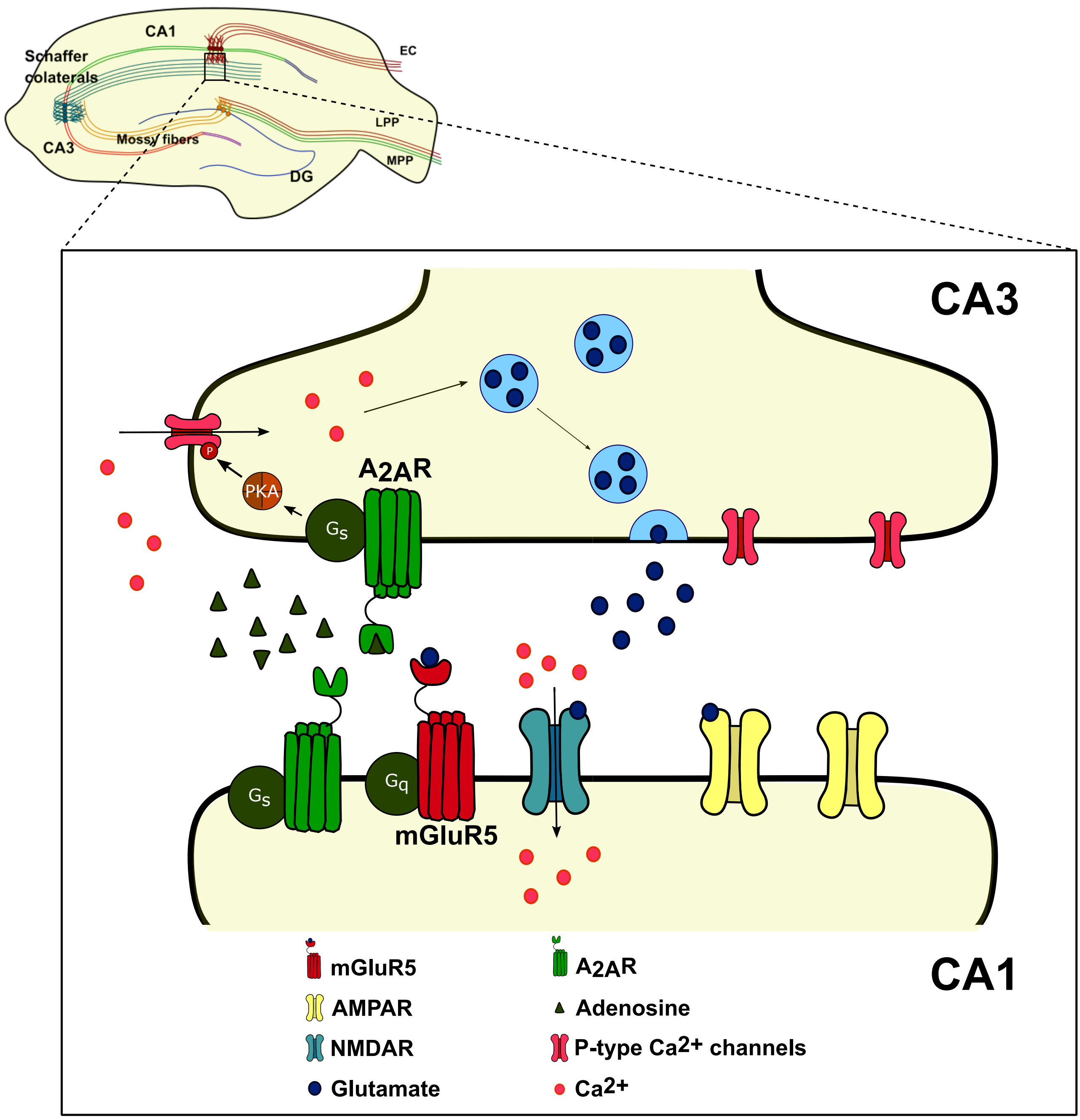

Supplementary Figure 5. Summary diagram of the $A_{2A}R$/NMDAR/mGluR5 interplay in age-related synaptic dysfunction in the hippocampus. Pathophysiological levels of $A_{2A}R$, mainly pre-synaptic, increases glutamate release likely by inducing Ca^{2+} uptake(29) and PKA-dependent Ca^{2+} currents through P-type Ca2+ channels in the presynaptic CA3 neurons(88) that project onto the CA1 pyramidal cells. Postsynaptically, a NMDAR overactivation drives an increase in Ca^{2+} influx, leading to a dysregulation in Ca^{2+} intracellular levels. This $A_{2A}R$-mediated NMDAR overactivation is dependent on mGluR5, since mGluR5 blockade prevents the $A_{2A}R$-mediated LTD-to-LTP shift and the NMDAR aberrant contribution in basal transmission. Accordingly, upon activation by glutamate release, preferentially by strong synaptic activation, mGluR5 increase NMDAR-mediated Ca^{2+} currents(65). Previous studies also hinted at a possible $A_{2A}R$-NMDAR crosstalk, since $A_{2A}R$ can control expression(50,101), recruitment(51) and the rate of desensitization(52) of NMDAR. We and others have provided compelling evidence of an $A_{2A}R$-mGluR5 synergistic interaction in the modulation of NMDAR-mediated effects(52,100,101,103). Thus, mGluR5 is a likely candidate to act as a switch between $A_{2A}R$ and NMDAR, by sensing glutamate and translating it into NMDAR overactivation. Furthermore, a putative post-synaptic $A_{2A}R$-mGluR5 direct interaction may also exist and increase NMDAR activation, as previously demonstrated(52, 89, 92), in which $A_{2A}R$ and mGluR5 can directly interact and regulate NMDAR activity(52,100).

Suplementary Figure 6

Figure 1a

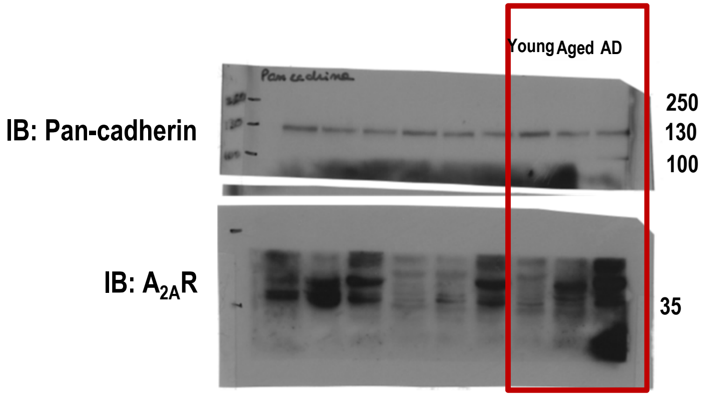

Supplementary Figure 4f

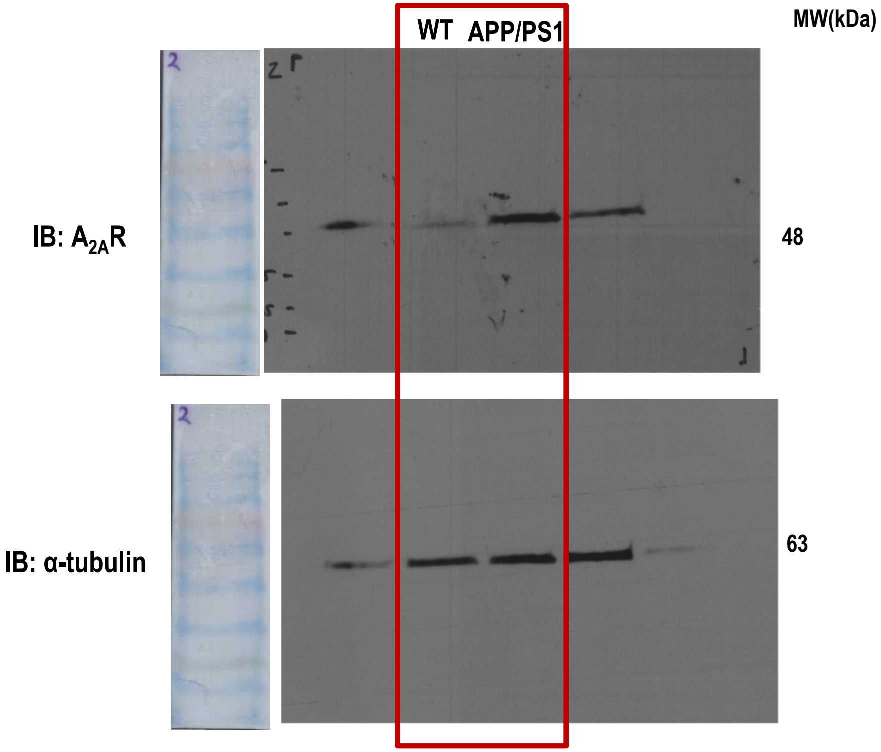

Figure 1h

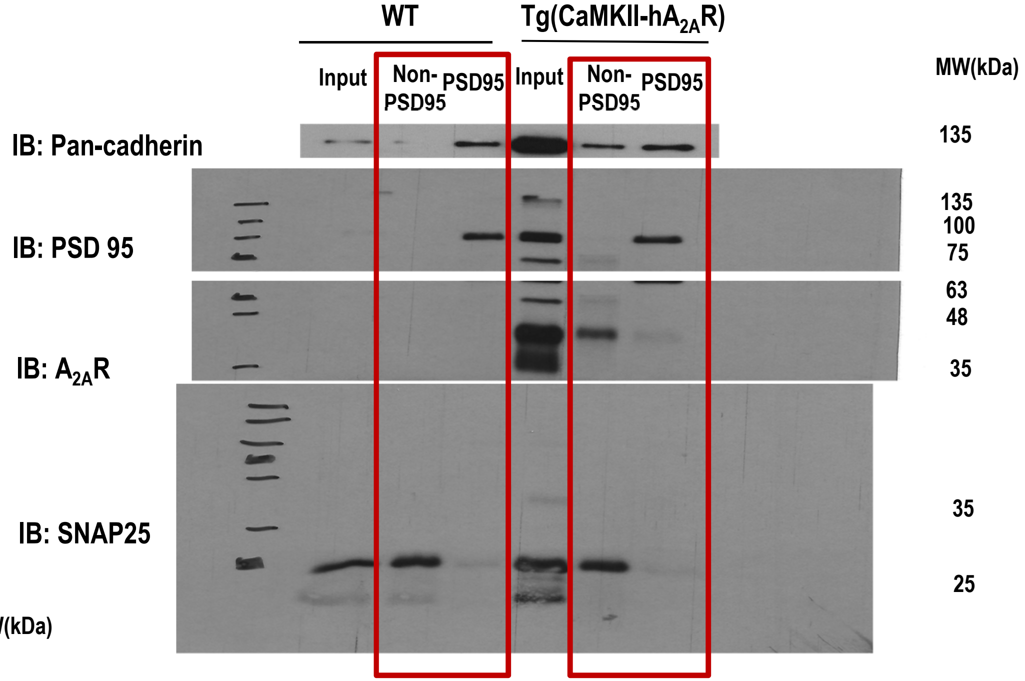

Molecular weight markers

ThermoFisher Scientific #26619

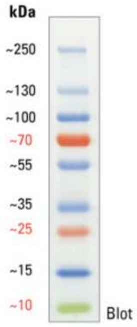

NzyTech, MB09002

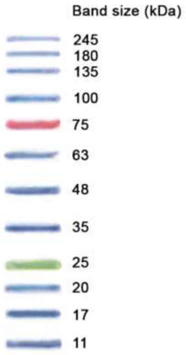

Supplementary Figure 6. Uncropped versions of representative western blot images from **Figure 1a**, **1h** and **Supplementary Figure 4f**. Some membranes were cut prior to antibody staining to allow for simultaneous detection of proteins running at different sizes on the same membrane without reprobing. Representative membranes with the molecular weight markers (MWM) are also illustrated.

Supplementary Video 1. $A_{2A}R$ activation increases intracellular Ca^{2+} levels in primary neuronal cultures transfected with Venus-$A_{2A}R$ construct. To investigate whether $A_{2A}R$-mediated NMDAR hyperactivation disrupted Ca^{2+} signaling, we measured variations in intracellular calcium concentrations ($[Ca^{2+}]i$) in primary neuronal cultures transfected with $A_{2A}R$. Application of the $A_{2A}R$ agonist CGS 21680, 30 nM, elevated intracellular Ca^{2+} levels in Venus-$A_{2A}R$ transfected neurons, whereas in non-transfected neurons lower changes in fluorescence were detected. Such increase in Ca^{2+} influx is prevented by NMDAR (AP5, 50 μM, **Figure 4d,h**) or $A_{2A}R$ (SCH 58261, 50 nm, **Figure 4e,i**) or mGluR5 (MPEP, 5 μM, **Figure 4f,j**) acute blockade.
